# Multi-dimensional evaluation of response to salt stress in wheat

**DOI:** 10.1371/journal.pone.0222659

**Published:** 2019-09-30

**Authors:** Said Dadshani, Ram C. Sharma, Michael Baum, Francis Chuks Ogbonnaya, Jens Léon, Agim Ballvora

**Affiliations:** 1 INRES Plant Breeding, Rheinische Friedrich-Wilhelms-University, Bonn, Germany; 2 International Center for Agricultural Research in the Dry Areas (ICARDA), Tashkent, Uzbekistan; 3 International Center for Agricultural Research in the Dry Areas (ICARDA), Rabat, Morocco; 4 Grains Research and Development Corporation, Barton, ACT, Australia; Institute of Genetics and Developmental Biology Chinese Academy of Sciences, CHINA

## Abstract

Soil salinity is a major threat to crop production worldwide. The global climate change is further accelerating the process of soil salinization, particularly in dry areas of the world. Increasing genetic variability of currently used wheat varieties by introgression of exotic alleles/genes from related progenitors’ species in breeding programs is an efficient approach to overcome limitations due to the absence of valuable genetic diversity in elite cultivars. Synthetic hexaploid wheat (SHW) is widely regarded as donor of favourable exotic alleles to improve tolerance against biotic and abiotic stresses such as salinity stress. In this study, synthetic backcross lines (SBLs) winter wheat population “Z86”, derived from crosses involving synthetic hexaploid wheat Syn86L with German elite winter wheat cultivar Zentos, was evaluated for salinity tolerance at different developmental stages under controlled and field conditions in three growing seasons. High genetic variability was detected across the SBLs and their parents at various growth stages under controlled as well as under salt stress field trials. Greater performance of Zentos over Syn86L was detected at germination stage across all salt treatments and with respect to shoot dry weight (SDW) and root dry weight (RDW) at seedling stage. Whereas for the root length (RL) and the shoot length (SL) Syn86L surpassed the elite cultivar and most of the progenies. Our experiments revealed for almost all traits that some genotypes among the SBLs showed higher performance than their parents. Furthermore, positive transgressive segregations were detected among the SBLs for germination at high salinity levels, as well as for RDW and SDW at seedling stage. Therefore, the studied Z86 population is a suitable population for assessment of salinity stress on morphological and physiological traits at different plant growth stages. The identified SBLs provide a valuable source for genetic gain through recombination of superior alleles that can be directly applied in breeding programs for efficiently breeding cultivars with improved salinity tolerance and desired agronomic traits.

## 1. Introduction

Salinity is a major threat to agricultural productivity worldwide and presents a tremendous challenge for food security [1]. More than one billion hectares of land, accounting for approximately 25% of the global land area, is affected by salinity. Due to natural salinization or unsuitable irrigation practices, this area is increasing by up to 10 million hectares of land every year [2]. Bread wheat (*Triticum aestivum* L. ssp. *aestivum*) is one of the most important crops contributing about 20% of the total dietary calories and proteins worldwide [3].

However, soil salinity is a major constraint for wheat production in many parts of the World affecting yield losses up to 60% [4] and causing food insecurity. In many arid and semi-arid areas of the World where wheat is the main staple food, soil salinity is intensifying the enduring food deficiency [5]. Despite intensive efforts, little success has been achieved in breeding salinity tolerant wheat varieties [6]. That is because salinity tolerance is a highly complex quantitative trait involving plant-specific morphological, physiological and metabolic processes regulating mechanisms to tolerate salinity stress. These mechanisms are generally categorized as osmotic tolerance, exclusion of toxic ions and tissue tolerance [7–10].

Osmotic tolerance involves all physiological adjustments in plants by production and/or allocation of osmoprotectants such as amino acids (e.g. proline) and sugars [11], selective preferential uptake of K^+^ and mechanisms of translocation of K^+^ in shoots by diverse K^+^ specific channels and transporters. The mechanism of exclusion is mainly to minimize the amount of toxic Na^+^—in the cytoplasm of roots and shoots and maintaining high K^+^/Na^+^ ratio [12]. If the salt concentration in leaves is high, plants with capability of tissue tolerance minimize the concentration of Na^+^ in cytoplasm and thus avoiding detrimental effects on cell metabolism by sequestration of large amount of salts in vacuoles and other cellular compartments [9, 13, 14]. During the last decades, research was mainly focused on the Na^+^ exclusion theory and maintenance of K^+^/Na^+^ ratio as the main trigger of salinity tolerance [15]. However, recent studies indicate higher impact of osmotic tolerance and tissue tolerance as main components of salinity tolerance [16, 17]. In general, plants respond to salinity stress with a combination of the above-mentioned mechanisms to persist the osmotic and ionic stress imposed by salinity stress.

Reduction of dehydration is an essential approach of plants to overcome lower osmotic potential in the rhizosphere. Beside establishment of waters channels like aquaporins [18] to facilitate the uptake of water, plants regulate stomatal conductance to reduce water loss by transpiration. However, stomatal closure inhibits the photosynthetic activity of plants consequently enhancing the overproduction of reactive oxygen species (ROS), and, finally, reducing plant growth and yield [19, 20]. Therefore, salinity stress leads to an energy problem of plants where the photosynthetic capacity of the plant will no longer be able to supply the carbohydrate requirement of young leaves, which further reduces their growth [8]. Therefore, plants that maintain their photosynthetic activity under salt stress condition are recognized as salt tolerant ones [21]. Whereas most studies investigated the chronic (long-term) effect of salinity stress on plants at seedling stage, only a few studies focused on the acute (short-term) effect of salinity stress on plants. In this context, Kawasaki et al. [22] and Geilfus et al. [23] detected genotypic variation of photosynthetic parameters of genotypes of rice and faba beans, respectively, shortly after exposed to salinity stress.

Despite some success in identification of genes that confer salinity tolerance in model plants, such as *Arabidopsis thaliana* (L.) and also complex plants with polyploid genomes, like hexaploid wheat under controlled environmental conditions, little success has been achieved in confirming this research outcome under field conditions [13, 16, 24, 25]. The reason for this is that most studies were conducted under controlled environmental conditions and not under natural field conditions with multiple environmental effects and that the focus was laid on distinct growth stages and not the entire life cycle of plants [26, 27]. However, due to spatial and temporal inhomogeneity of soil salinity, screening a large number of genotypes under field conditions with natural salinization is challenging. Furthermore, under field conditions, differentiation of distinct response of plants to salt stress is difficult as salinity stress occurs frequently with other abiotic stresses such as drought and heat stress. Though, field trials under natural salinization are essential assessment approaches for authentication of results obtained from testing systems under controlled environmental conditions.

Moreover, plants diversely respond to salinity stress at different developmental stage [28, 29]. Generally, plants are more sensitive at early growth stages and less at seedling stage [28, 30] and salt stress during early reproductive stage has high impact on grain yield [31, 32]. However, germination rate is commonly used to assess salinity tolerance at germination stage [33]. Whereas biomass production and grain yield are frequently investigated at seedling and maturity stage, respectively [34, 35].

For many years, breeding for salinity tolerance was not a priority for wheat breeders [36]. Lack of precise characterization of physiological and morphological traits related to salinity stress at different growth stages and low genetic variability of currently available wheat varieties is one of the main reasons for limited success in breeding salt-tolerant wheat varieties [37]. Only a few studies were investigating salinity stress of wheat plants at germination stage, seedling stage, and, furthermore, under field conditions. Among them, Oyiga et al. [38] were characterizing the salinity tolerance of an association panel of wheat germplasm under various salt stress conditions at different growth stages.

Genetic variability is essential for efficiently breeding salinity tolerant wheat cultivars. Breeding new cultivars with adaptation to harsh environmental conditions by introgression of favourable exotic alleles into modern cultivars is a widely accepted approach to overcome the genetic bottleneck imposed by domestication and modern breeding process [39, 40]. However, extensive screening approaches of a large number of genotypes led to the detection of only a few landraces like Candeal (Spain), Kharchia (India) and Shorawaki (Pakistan) possessing a certain degree of salinity tolerance [41–44]. And, despite all breeding efforts, little success was achieved in breeding salinity tolerant wheat cultivars using wild relatives due to the linkage drag associated with unfavourable characteristics such as lodging, low baking and threshing quality [6, 13, 45].

However, neoallopolyploids like synthetic hexaploid wheat (SHW) offer enormous genetic variability. Particularly, SHW derived from hybridizations between *Aegilops tauschii* and *Triticum turgidum* spp. *dicoccoides*, the wild tetraploid progenitor of *Triticum diccocum*, harbour a large number of unexploited exotic alleles [46].

Furthermore, the use of synthetic backcross-derived lines (SBLs), based on the cross between SHW as a donor of exotic alleles and modern wheat cultivars as a recurrent parent, are widely used for mapping genomic regions linked to specific traits, detection of epistatic interactions and identification of germplasm possessing improved tolerance/resistance to biotic and abiotic stresses [47, 48]. Thus, SBLs based on near-isogenic lines (NILs) or recombinant-inbred lines (RILs), carrying small introgressions of the exotic parent, allow detection of beneficial alleles that are ideal for pyramiding of desirable traits, reducing the chance of linkage drag.

However, SHW and SBLs show high phenotypic variation with respect to diverse traits. According to Dreisigacker [49], this phenotypic diversity is mainly based on (a) the genetic variation of the *Ae*. *tauschii* accession parent that was selected as progenitor, (b) modification of gene expression caused by genomic changes during artificial hybridization and (c) changing epistatic interactions on the background of introduced homeologous chromosomes of A and B genomes.

To date, no scientific report is available assessing salinity tolerance of a synthetic derived segregating population wheat at different growth stage. The main objective of the present study was to characterize the SBLs obtained from a cross between the German elite cultivar Zentos and the synthetic genotype Syn86L that were evaluated for salinity tolerance at germination, seedling and maturity stage and to identify traits related to salinity tolerance. Furthermore, to identify genotypes among the SBLs with higher salinity tolerance that can be utilized to breed salt tolerant varieties.

## 2. Materials and methods

### Plant material

A winter wheat population of 151 SBLs, denoted as “Z86” derived from parents Zentos and Syn86L was used [50]. Briefly, this population was constructed according to the advanced backcross (AB) strategy described by Tanksley and Nelson [51] by crossing the German elite winter wheat cultivar “Zentos” with the synthetic hexaploid wheat “Syn086L”. The elite parent Zentos was registered at Bundessortenamt (the Federal Plant Varieties Office of Germany) in 1989 as a high yielding variety (Bundessortenamt 2016). The synthetic parent, Syn086L, was produced by Lange and Jochemsen [46] by crossing wild emmer (*Triticum turgidum* spp. *dicoccoides*; accession number G4M-1M) as donor for AABB genome and *Aegilops tauschii* (accession number Gat-525) as the donor of DD genome. Since the emasculation of *Aegilops tauschii* was more complicated than of wild emmer, *Aegilops tauschii* was acting as the male parent and wild emmer as the female parent, respectively. After two times of backcrossing with the recurrent elite parent Zentos, the population was derived to BC2F_3:7_ by several steps of selfing and bulk propagation. Genetically, each line of the Z86 population had small chromosomal introgressions of the synthetic parent in the background of the elite parent [50]. Seeds of the elite cultivar Zentos were kindly provided by Syngenta Seeds GmbH (Bad Salzuflen, Germany).

### Phenotypic analysis

#### Germination experiments

Germination tests were carried out following the protocol described by Mano and Takeda [33]. Seeds of the testing population were surface sterilized with 70% ethanol for one minute followed by three times rinsing with deionized water. Ten seeds of equal size were placed on filter paper (C160; Munktell & Filtrak GmbH, Germany) laying in crystal clear rectangular boxes (V3-92; Licefa GmbH & Co. KG, Germany). Salt treatments were applied by watering of seeds with defind salt concentrations. The applied salt concentrations were 50, 100, 150, 200 and 250 mM NaCl (CAS 7647-14-5, for analysis, PanReac AppliChem GmbH, Germany) and 50, 100 and 150 mM Na_2_SO_4_ (CAS 7757-82-6, for analysis, PanReac AppliChem GmbH, Germany) respectively, and, whereas, the control conditions did not contain additional salt [52]. The seeds and the resulting seedlings were incubated for 10 days in the climate chamber at 20±2°C with 50±5% humidity at 12h light (200 μmol m^-2^ s^-1^) and 12h dark periods per day. The germination rate of seeds was scored according to the germination scoring scheme from Badrize et al. [53] adapted from Mano et al. [54], whereas 0 was given for no germination and 9 for seeds producing leaves longer than 6 cm from the coleoptile.

#### Hydroponic experiment

At seedling stage, the plants were tested in hydroponic system with 100 mM NaCl or 100 mM Na_2_SO_4_ and under control conditions with no additional salt application (Figure A in S1 File). For this purpose, seeds were germinated without application of salts. After 8 days, uniform plantlets were selected to be transferred into the hydroponic system consisting of 12 light-tight polypropylene boxes with 170 dm^3^ capacity (EG 86/42 HG by Auer Packaging, Germany). The boxes were covered with light-tight styrodur panels (BASF, Germany). Each box was continuously aerated by four adjustable air diffusers (Eheim 4002650, Germany) supported by electric air pumps. Each panel was prepared with 54 holes where non-hygroscopic sponges were holding the plants on the solution surface. The boxes were filled with pure tap water with an EC value of 0.08 mS/cm [55]. Nutrient solutions were added to the water according to Shavrukov et al. [56]. After eight days of adaptation in the hydroponic system, the salinity level of the salt treated boxes was increased incrementally in three days by adding 33.3 mM of NaCl and Na_2_SO_4_, respectively, until reaching the final concentration of 100 mM. The specifity of the utilized salts are discribed in the section *Germination experiments*.

Every second day, the pH was adjusted between 6.2 and 6.5 by adding HCl or NaOH using portable pH-Meter (SG2-FK SevenGO, Mettler Toledo, Switzerland). The solution was renewed every 9 days. The hydroponic boxes were placed during the testing period in the greenhouse with 20°C at day and 12°C at night and 12h/12h light/dark period per day. Shoot (SL) and root lengths (RL) were measured at stress initiation, as well as 9 and 16 days after stress initiation (DAS). In addition, at harvest (16 DAS), root and shoot weights were measured. To estimate the dry weight mass, the plant material was weighted after drying for three days at 65°C. Calcium, potassium and sodium concentrations in third leaves were measured by the Atomic Absorption Spectrometer (AAS) AAnalyst 200 (Perkin Elmer, USA) following the method described by Madejczyk and Baralkiewicz [57].

#### Field experiments

The 151 lines of the Z86 population were evaluated under field conditions with natural salinization (saline and non-saline) in Karshi (Uzbekistan; 38°52′N 65°48′E) in three growing seasons in the years 2010 to 2013. The soil type was silty clay with a mixture of chloride-sulphate salts (sulphate/chloride ratio 1.9 to 4.6). Due to the natural soil salinization, the intensity of soil salinity of the experimental site was heterogeneous. The EC value of the non-saline plots ranged between 0.62 and 1.34 mS/cm whereas the EC value of saline plots was between 2.3 and 3.8 mS/cm. The weather details during the experimental period are presented in Figure B in S1 File. The plots were arranged according to Alpha Lattice design with three replications. Among the measured traits were days to heading (DHD), plant height (PH), peduncle length (PL), spike length (SPL), number of spikelets per spike (SpS), 1000 kernel weight (TKW) and grain yield per m^2^. The agronomic traits were measured according to the procedure described by Sharma et al. [58]. Near-infrared reflectance spectroscopy (NIR) with Diode Array 7250 NIR analyser (Perten Instruments, Inc., USA) was used to analyse grain quality parameters, including protein and starch content.

### Data analysis and evaluation

The Stress Tolerance Index (STI) was calculated according to Fernandez et al. [59].

$$STI=\frac{{Trait}_{control}*{Trait}_{treatment}}{{{(Trait}_{av;\;control})}^{2}}$$ Eq 1

Where Trait_control_ stands for the value of the trait under controlled condition and Trait_treatment_ stands for the value of the parameter under treatment. Trait_av;control_ stands for the population average under control conditions.

The broad-sense heritability (H^2^) was estimated using Restricted Maximum Likelihood (REML) method described in Holland et al. [60].

$$H^{2}=\frac{V_{G}}{V_{G}+\frac{V_{G*T}}{t}+\frac{V_{E}}{t*r}}$$ Eq 2

where V_*G*_ genetic variance, *T* treatment, V_*E*_ error term, *t* and *r* denote the number of treatments and the number of replications, respectively. Mid-parental transgressive segregation (MTS) was calculated as following:

$$MTS={\bar{x}}_{P}+{2\sigma }_{P}$$ Eq 3

where ${\bar{x}}_{P}$ and 2σ_*P*_ are the mean and the standard deviation, respectively, of both parents for a specific trait. The Best-parental transgressive segregation (BTS) was calculated according to:

$$BTS={\bar{x}}_{BP}+{2\sigma }_{BP}$$ Eq 4

where ${\bar{x}}_{BP}$ and σ_*BP*_ are the mean and the standard deviation, respectively, of the parent with the highest value for the specific trait.

PROC GLM of SAS 9.4 [61] was utilized to apply the regression method for the analysis of repeated-measures of data over time by treating time as a quantitative regression variable [62]. Accordingly, the effects and interactions of genotype (G), treatment (T) and under the time course temporal response (Z) were calculated using application of orthogonal polynomial transformation option (Eq 5). In this model, genotype and treatment were set as independent variables. The different time points were regarded as a within-subjects factor, where every time point was regarded as one single measurement.

$$Y_{ijx}=\mu +T_{i}+G_{j}+Z_{x}+T_{i}*G_{j}+G_{j}*Z_{x}+T_{i}*Z_{x}+T_{i}*G_{j}*Z_{x}+\varepsilon_{ijx}$$ Eq 5

where Y_*ijx*_ is the phenotypic value; μ, general mean; *T*_*i*_, the fixed effect of *i*-th treatment; *G*_*j*_, the fixed effect of *j*-th genotype; *Z*_*x* (*x* = 1 …n)_, the time point; *T*_*i*_ * *G*_*j*_, the fixed interaction effect of *i*-th treatment with *j-*th genotype; *G*_*j*_ * *Z*_*x*_, the random interaction effect of of *j*-th genotype with of *x*-th time point; *T*_*i*_ * *Z*_*x*_, the effect of interaction of *i*-th treatment with *x*-th time point; *T*_*i*_ * *G*_*j*_ * *Z*_*x*_, the fixed interaction effect of *i*-th treatment and with *j-*th genotype and with *x*-th time point; and *ε*_*ijx*_, the random errors (the residual).

## 3. Results

Several experiments at different plant growth stages were conducted to characterize the diverse effects of salt stress at specific developmental stages of the SBLs of the Z86 winter wheat population and their parents Zentos and Syn86L.

### Phenotypic characterization at germination stage

**Fig 1:**
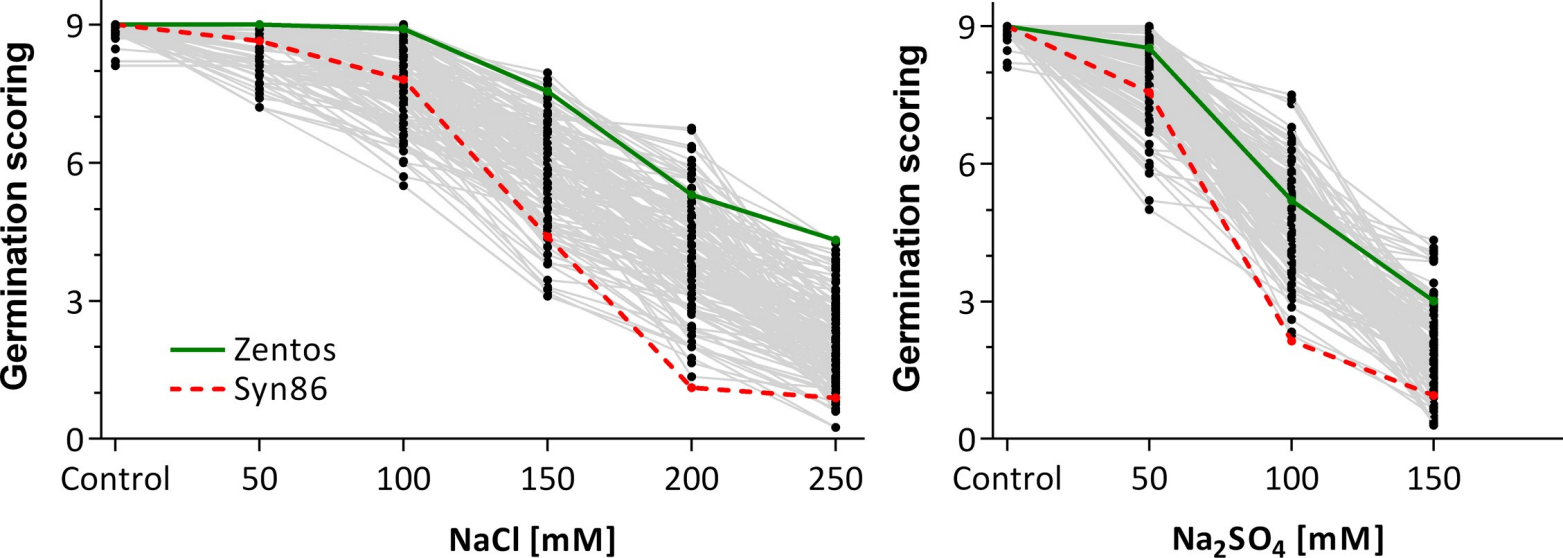
Overview of the germination scores (y-axis) of the 151 SBLs of the Z86 population and parent Zentos (green) and Syn86L (red) at different salt concentrations (x-axes) of NaCl (A) and Na_2_SO_4_ (B); dots represent the genotypes and are connected with lines for a better overview.

Germination tests revealed high genetic diversity in response to the tested salt treatments with reduced leaf and root development under higher concentrations (Fig 1). Irrigation with saline water reduced the germination scores of all tested genotypes. At equimolar concentrations, the negative effect of Na_2_SO_4_ on germination score of tested genotypes was higher than NaCl (Tables 1 and 2). The parents of the Z86 population (Syn86L and Zentos) showed contrasting germination scores at almost all tested salt treatments except for 50 mM NaCl concentration. The germination scores of the recurrent parent Zentos were for both salt types and all concentrations higher than that of the most of its progenies and the synthetic parent Syn86L. At high salt concentrations, the synthetic parent was among the genotypes with the lowest germination score. At 200 mM NaCl, 15% of the SBLs showed Mid-parental transgressive segregation (MTS), whereas only two genotypes, namely, WW35-78 and WW42-42, showed MTS at 250 mM NaCl. At 100 mM Na_2_SO_4_, 8% of the SBLs showed MTS and at 150 mM Na_2_SO_4_ genotype WW34-48 was the only line showing MTS. Significant genotype by treatment interaction effects among the SBLs were detected for all salt treatments except for the lowest NaCl concentration of 50 mM. The broad-sense heritability (H^2^) for the germination score with different salt treatments was between 19 and 72%, whereas low H^2^ values correspond to control and low salt concentrations and higher H^2^ values correspond to higher salt concentrations.

**Table 1:** Descriptive statistics of germination scores of the Z86 population (SBLs and parents) for different salt treatments.

Treatment		Z86 parents		SBL average	SD	H^2^ [%]
		Zentos	Syn86L			
**Control**		9.0^a^	9.0^a^	8.9^a^	0.3	32.4
**NaCl** **[mM]**	50	9.0^a^	8.6^ab^	8.6^b^	0.5	22.3
	100	8.9^a^	7.8^b^	7.8^b^	1.1	31.2
	150	7.5^a^	4.4^c^	6.0^b^	1.6	46.7
	200	5.3^a^	1.1^c^	4.3^b^	1.2	62.5
	250	4.3^a^	0.9^c^	2.3^b^	0.7	70.7
**Na_2_SO_4_ [mM]**	50	8.5^a^	7.5^b^	7.9^ab^	1.0	19.2
	100	5.2^a^	2.2^c^	4.9^b^	1.0	48.2
	150	3.0^a^	0.9^c^	2.0^b^	1.1	71.8

SBLs = synthetic derived backcross lines of the Z86 population, SD = standard deviation, H^2^ = broad sense heritability, different superscript letters indicate significant differences between the treatments and the groups (SBLs, Zentos and Syn86L) according to Tukey Honestly Significant Difference (HSD) with p ≤ 0.05.

**Table 2:** Analysis of variance of germination scores of the SBLs of Z86 population explaining genotype, treatment and genotype by treatment effects.

Treatment		Genotype (G)		Treatment (T)		G*T	
		*F*-values	df	*F*-values	df	*F*-values	df
**Control**		1.96***	150				
**NaCl** **[mM]**	50	1.86***	150	51.97***	1	1.02^ns^	150
	100	2.36***	150	585.35***	1	1.98***	150
	150	3.63***	150	4103.84***	1	3.53***	150
	200	6.00***	150	13893.30***	1	5.67***	150
	250	8.24***	150	52010.20***	1	7.82***	150
**Na_2_SO_4_ [mM]**	50	1.71***	150	460.52***	1	1.53***	150
	100	3.80***	150	8599.17***	1	3.85***	150
	150	8.63***	150	57544.2***	1	8.47***	150

*** p ≤0.001

ns = not significant, df = degree of freedom

Among the tested genotypes, SBL WW35-84 and WW42-42 showed the highest salinity tolerance under 250 mM NaCl with germination scores of 4.25. Under the highest tested Na_2_SO_4_ concentration (150 mM), SBL WW42-31 showed the lowest germination score value with 0.3 whereas the most tolerant genotype at this salt treatment level, line WW34-48, was scored with 4.3. This genotype showed the highest germination score (6.7) among all genotypes under 200 mM NaCl, but moderate tolerance with germination score of 1.15, when the salt concentration was increased to 250 mM NaCl. Overall, as shown in the correlation matrix (Table 3), the correlations among the tested salt treatments was low to moderate, with higher values between the different concentrations of NaCl and low correlations among the Na_2_SO_4_ concentrations.

**Table 3:** Pearson's correlation coefficient (r) for germination scores at different salt stress treatments.

			NaCl [mM]					Na_2_SO_4_ [mM]		
		Control	50	100	150	200	250	50	100	150
**Control**		1								
**NaCl [mM]**	**50**	0.230**	1							
	**100**	0.139*	0.565**	1						
	**150**	-0.107	0.303**	0.496**	1					
	**200**	-0.058	0.244**	0.321**	0.625**	1				
	**250**	0.096	0.194**	0.209**	0.198**	0.473**	1			
**Na**_**2**_**SO**_**4**_ **[mM]**	**50**	0.064	0.203**	0.280**	0.186*	0.350**	0.395**	1		
	**100**	0.202**	0.191**	0.277**	0.392**	0.299**	0.302**	0.355**	1	
	**150**	-0.199**	0.112	0.403**	0.417**	0.375**	-0.001	0.139*	0.160*	1

Significance levels p values

* p ≤ 0.05

** p ≤ 0.01; *** p ≤ 0.001

Moreover, as the correlation between the two salt types at the same molar concentration was high to moderate, it was reduced by increasing the molar concentration of the corresponding salt type.

### Phenotypic characterization at seedling stage

**Fig 2:**
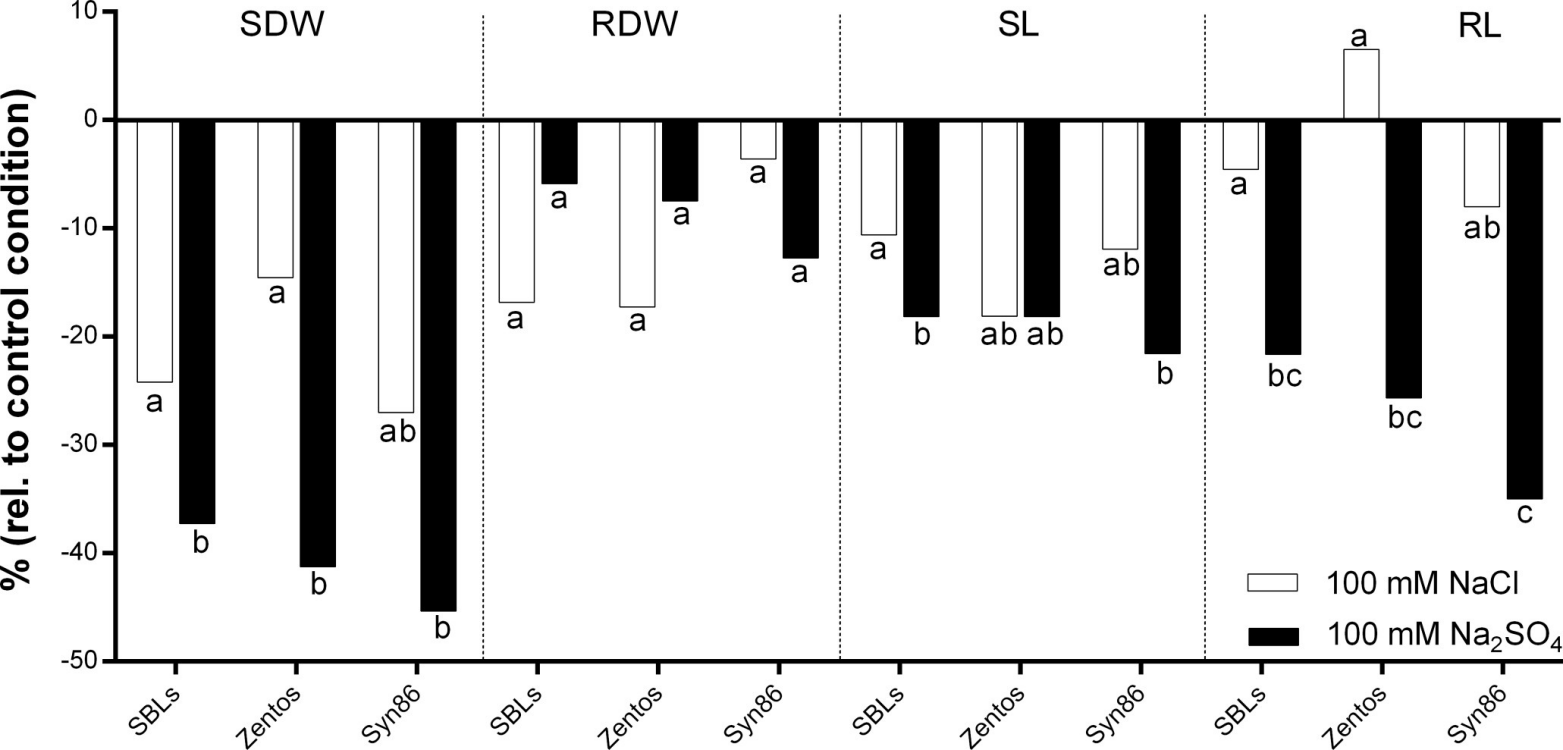
Overview of shoot dry weight (SDW), root dry weight (RDW), shoot length (SL) and root length (RL) of Zentos, Syn86L and Z86 population mean under 100 mM NaCl and Na_2_SO_4_ relative to control conditions; different subscript letters indicate significant differences between the treatments and the groups (SBLs, Zentos and Syn86L) according to Tukey Honestly Significant Difference (HSD) with p ≤ 0.05.

To characterize the salinity tolerance of the AB-lines of the Z86 population and their parents Zentos and Syn86L at seedling stage, the genotypes were tested under 100 mM concentration of NaCl and Na_2_SO_4_, respectively, in comparison to control conditions. Fig 2 shows the effect of salt treatments, on SDW, RDW, SL and RL for the SBLs and their parents. For most analysed traits, the deleterious effect of Na_2_SO_4_ was higher than the equimolar concentration of NaCl. However, RDW of the parent Zentos and the population average showed stronger reduction under treatment with NaCl. Syn86L plants exhibit higher reduction in RL under the both, 100 mM NaCl and Na_2_SO_4_. However, their roots were in average +31% and +23% longer than roots Zentos plants when grown in 100 mM NaCl and Na_2_SO_4_, respectively. Analysis of variance revealed significant treatment effects for all analysed traits, except for RDW. However, no significant genotype by treatment interactions were detected for RL at 100 mM Na_2_SO_4_ (Table 4).

**Table 4:** Analysis of variance explaining genotype, treatment and genotype by treatment interactions for the major traits (SDW, RDW, SL and RL) of the Z86 population measured at seedling stage under 100 mM of salt stress treatments.

	100 mM NaCl							100 mM Na_2_SO_4_						
	Genotype (G)		Treatment (T)		G*T		H^2^	Genotype (G)		Treatment (T)		G*T		H^2^
Parameter	F-values	df	F-values	df	F-values	df	[%]	F-values	df	F-values	df	F-values	df	[%]
**SDW**	1.29^ns^	150	95.09***	1	0.57^ns^	150	36.0	0.69^ns^	150	161.54***	1	0.30^ns^	150	39.3
**RDW**	1.07^ns^	150	38.86***	1	0.65^ns^	150	25.6	0.96^ns^	150	2.77^ns^	1	0.34^ns^	150	32.4
**SL**	1.12^ns^	150	64.88***	1	0.34^ns^	150	68.0	1.66***	150	611.02***	1	0.66^ns^	150	57.4
**RL**	2.06***	150	7.07***	1	0.50^ns^	150	73.5	1.45**	150	1.45**	1	0.47^ns^	150	62.3

SDW = shoot dry weight, RDW = root dry weight, SL = shoot length, RL = root length

significance levels of p

** p ≤0.01

*** p ≤ 0.001

ns = not significant

df = degree of freedom, H^2^ = broad sense heritability

**Fig 3:**
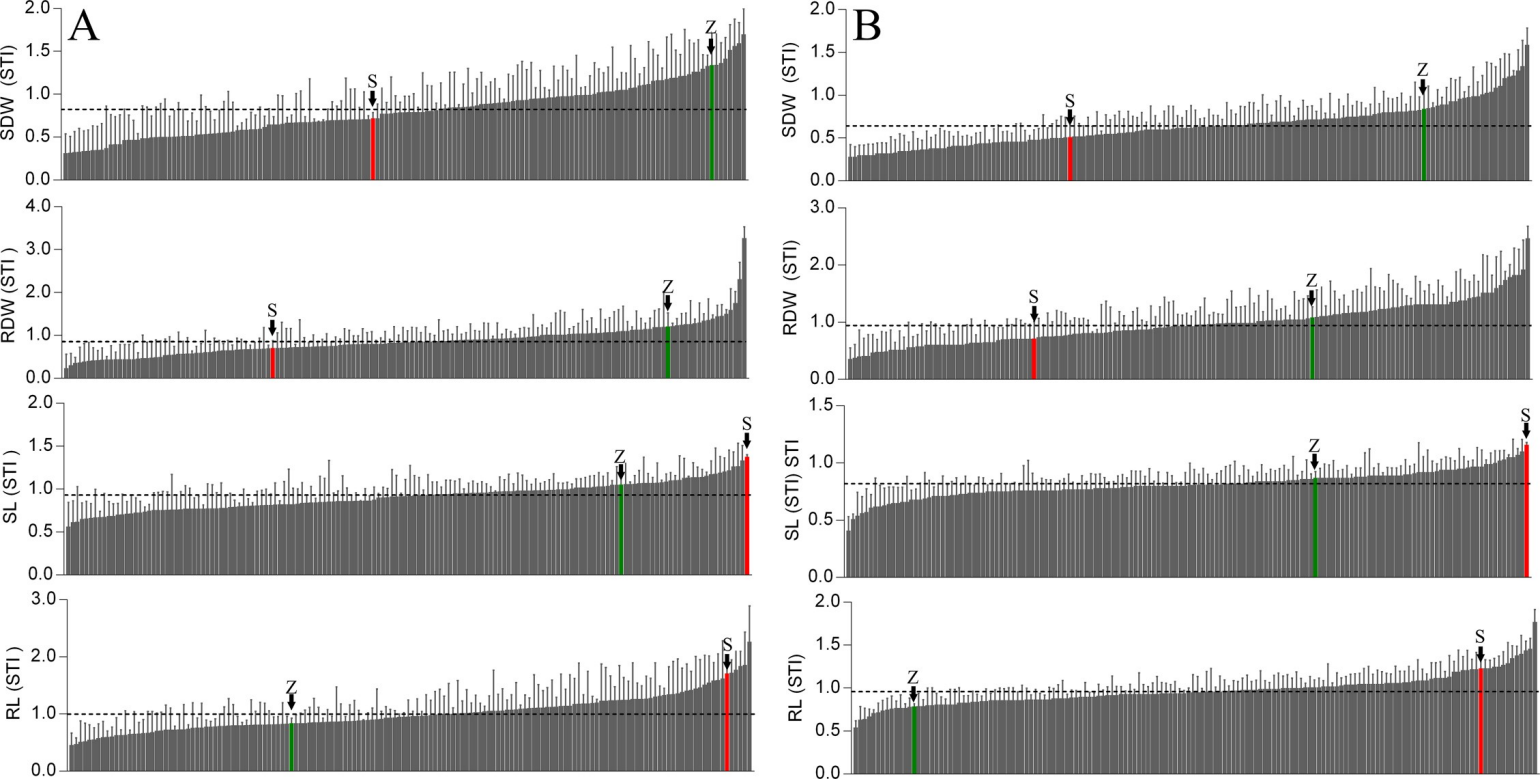
Bar plot of STI values of shoot dry weight (SDW), root dry weight (RDW), shoot length (SL), root length (RL) of the Z86 population and the parents Zentos and Syn86L tested in hydroponic systems with 100 mM NaCl (A) and 100 mM Na_2_SO_4_ (B); genotypes are sorted according to the increasing STI values (from left to right); the parents are highlighted with the letters S (Syn86L) and Z (Zentos); error bars indicate standard deviations; dashed lines indicate population means.

Comparing the STI values for the biomass parameters SDW and RDW revealed that the elite cultivar Zentos was outperforming the synthetic parent Syn86L and most of the progenies under both salt type used to induce stress (Fig 3). On the other hand, Syn86L showed higher salinity tolerance with respect to SL and RL in comparison to most of its progenies and Zentos. RDW of Zentos and its progenies were stronger reduced under NaCl treatment than under Na_2_SO_4_. Although Syn86L plants experienced a higher reduction in RL under 100 mM NaCl and Na_2_SO_4_, they had in average +31% and +23%, respectively, longer roots than Zentos plants. Under 100 mM NaCl, Zentos plants were producing longer roots (+6.5%) than under control conditions, even though their RDW was reduced by 20%. Among the SBLs, genotype WW40-40 and WW42-32 were showing consistently higher STI values for SDW compared to the population average at 100 mM NaCl with +110% and +93.9% and at 100 mM Na_2_SO_4_ treatment with +92% and +73.3%, respectively.

Mid-parental transgressive segregation (MTS) at sodium sulphate treatment was observed for SDW (three SBLS), RDW (six SBLs) and RL (18 SBLs). On the other hand, MTS at sodium chloride treatment was detected only for RDW (3 SBLs). Several SBLs were showing best-parental transgressive segregation BTS outcompeting the best-parent under 100 mM Na_2_SO_4_ for SDW (WW40-15, +8.5%), RDW (WW35-92 with 1% and 30%, respectively) and RL (WW35-93, +21%). Under NaCl treatment, only two SBLs were showing best-parent transgression with respect to RDW with WW40-40 (+17%) and WW38-20 (+66%).

Likewise, genotypes with the highest reduction of SDW under 100 mM sodium chloride also performed weak under sodium sulphate treatment. Genotype WW37-58 faced higher reduction of SDW with -58% and -53% under NaCl and Na_2_SO_4_ treatments, respectively. Only a few offspring had higher STI values for RL than Syn86L under both salt treatments, among them genotypes WW35-93 and WW43-45.

**Fig 4:**
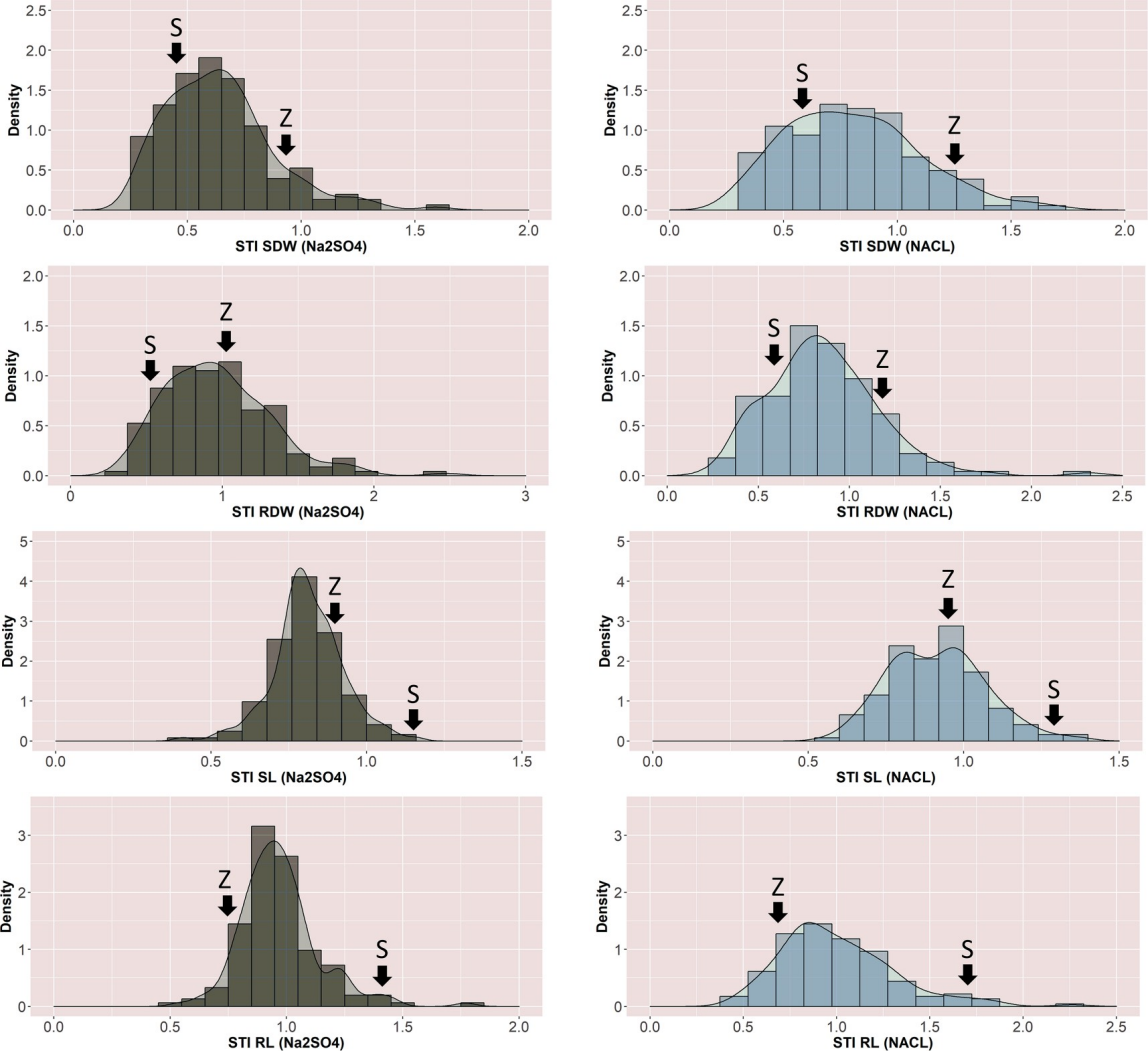
Distribution of STI (salinity tolerance index) values (x-axes) of main parameters measured at seedling stage of the Z86 population, SDW shoot dry weight, RDW root dry weight, SL shoot length, RL root length; the parents are highlighted with the letter S (Syn86L) and Z (Zentos).

In summary, corresponding to the germination test under different salt treatments, at equimolar concentration, the negative effect of Na_2_SO_4_ on SDW of the Z86 was higher than NaCl. The range of SDW among the Z86 population was reduced under 100 NaCl and further reduction of SDW was observed at treatment with 100 mM Na_2_SO_4_ (Fig 4 and Figure C in S1 File). SL was also reduced by both salt treatments whereas no significant difference in the detrimental effect of sodium sulphate and sodium chloride treatments were visible. Corresponding to the observation of SDW under different salt treatments, in average 100 mM Na_2_SO_4_ was inducing stronger reduction of RL than 100 mM NaCl. High correlation was observed between RDW and SDW under control conditions as well as both salt treatments. However, lower correlation was detected between RL and SL. The genetic variation of the described phenotypic traits among the SBLs were reduced under sodium chloride treatment and further reduction was observed under sodium sulphate treatment (Fig 4).

#### Analysis of root and shoot length in the temporal course of salinity stress at seedling stage

**Fig 5:**
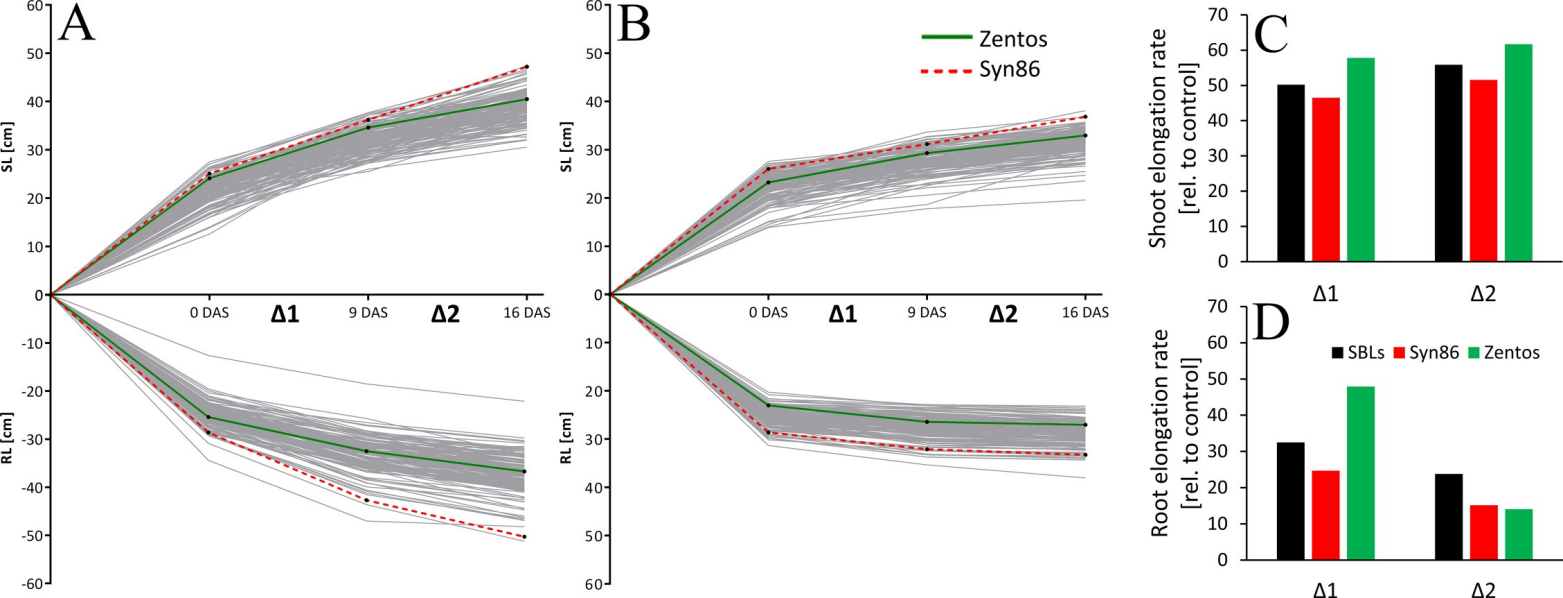
Overview of the average values of root and shoot length of the Z86 population under controlled (A) and salt stress conditions with 100 mM Na_2_SO_4_ (B) at 0, 9 and 16 DAS (days after stress initiation; Δ1 is the period between 0–9 DAS and Δ2 is the period 9–16 DAS); shoot elongation rate (SEL) and root elongation rate (RER) relative to control conditions are shown in (C) and (D), respectively; the parents are highlighted with the green solid line (Zentos) and red dashed line (Syn86L).

To investigate the chronic (long-term) effect of salt stress with 100 mM Na_2_SO_4_ on root and shoot traits, the RL and SL of the Z86 population, as well as the parents, were measured at three time points: 0, 9 and 16 DAS (Fig 5).

These measurements revealed stronger reduction of root elongation rates (RER) than shoot elongation rates (SER) at 100 mM Na_2_SO_4_ (Fig 5A and 5B). Furthermore, the reduction of SER (Fig 5C) was lower during Δ2 whereas stronger reduction of RER was detected during Δ2 (Fig 5D). Notably, during Δ1, RER of Zentos was less reduced than that of Syn86L and the population average. However, at Δ2, the RER of Zentos was facing stronger reduction comparing to Syn86L and the population mean.

Repeated measures analysis of variance (RMANOVA) was conducted for analysing the temporal effect of the interaction of genotype and treatment (100 mM Na_2_SO_4_) on RL and SL of the SBLs, including their parents. The sphericity, which is essential for RMANOVA, was not violated for both traits as the corresponding Greenhouse-Geisser Epsilon and Huynh-Feldt-Lecoutre Epsilon were not significant (Table 5).

**Table 5:** Main effects and interactions related to root and shoot length of Zentos and Syn86L under the temporal course of salinity stress with 100 mM Na_2_SO_4_.

	Shoot length					Root length				
Source	DF	*F*-value	*p*	Adj. *p*		DF	*F*-value	*p*	Adj. *p*	
				G - G	H-F-L				G - G	H-F-L
**Time**	2	156.18	<0.0001	< .0001	<0.0001	2	132.20	<0.0001	<0.0001	<0.0001
Genotype*Time	2	9.37	0.0003	0.0003	0.0003	2	12.47	<0.0001	<0.0001	<0.0001
Time*Treatment	2	35.28	<0.0001	< .0001	<0.0001	2	50.84	<0.0001	<0.0001	<0.0001
Genotype*Time* Treatment	2	2.90	0.0627	0.0635	0.0627	2	6.55	0.0026	0.0028	0.0026
**Error (Time)**	62					62				
Greenhouse-Geisser Epsilon 0.9866						Greenhouse-Geisser Epsilon 0.9866				
Huynh-Feldt-Lecoutre Epsilon 1.0533						Huynh-Feldt-Lecoutre Epsilon 1.0480				

Note: DF degree of freedom, F F-value, G-G Greenhouse-Geisser, H-F-L Huynh-Feldt-Lecoutre.

The genotype*treatment*time (G*T*Z) interaction was not significant for SL as well as for RL of the SBLs of the Z86 population. However, significant G*T*Z interaction was detected for the parents, Zentos and Syn86 for RL but for SL was not significant for Zentos and Syn86. Yet, for RL, significant genotype by treatment interaction was detected for the parents at Δ1, but not at Δ2 (Table 6).

**Table 6:** Repeated measures analysis of variance for contrast variables Δ1 (0–9 DAS) and Δ2 (9–16 DAS) for RL of Zentos and Syn86L exposed to 100 mM Na_2_SO_4_.

Contrast variables		Δ1 (0–9 DAS)		Δ2 (9–16 DAS)	
Source	DF	*F*-value	*p*	*F*-value	*p*
**Mean**	1	234.51	<0.0001	4.48	0.0424
**Genotype**	1	18.78	<0.0001	4.59	0.0400
**Treatment**	1	86.80	<0.0001	5.94	0.0207
Genotype* Treatment	1	11.65	0.0018	0.18	0.6739
**Error**	31				

#### Assessment of Na^+^, K^+^ and Ca^2+^ concentration in leaves under salinity stress and control condition

**Fig 6:**
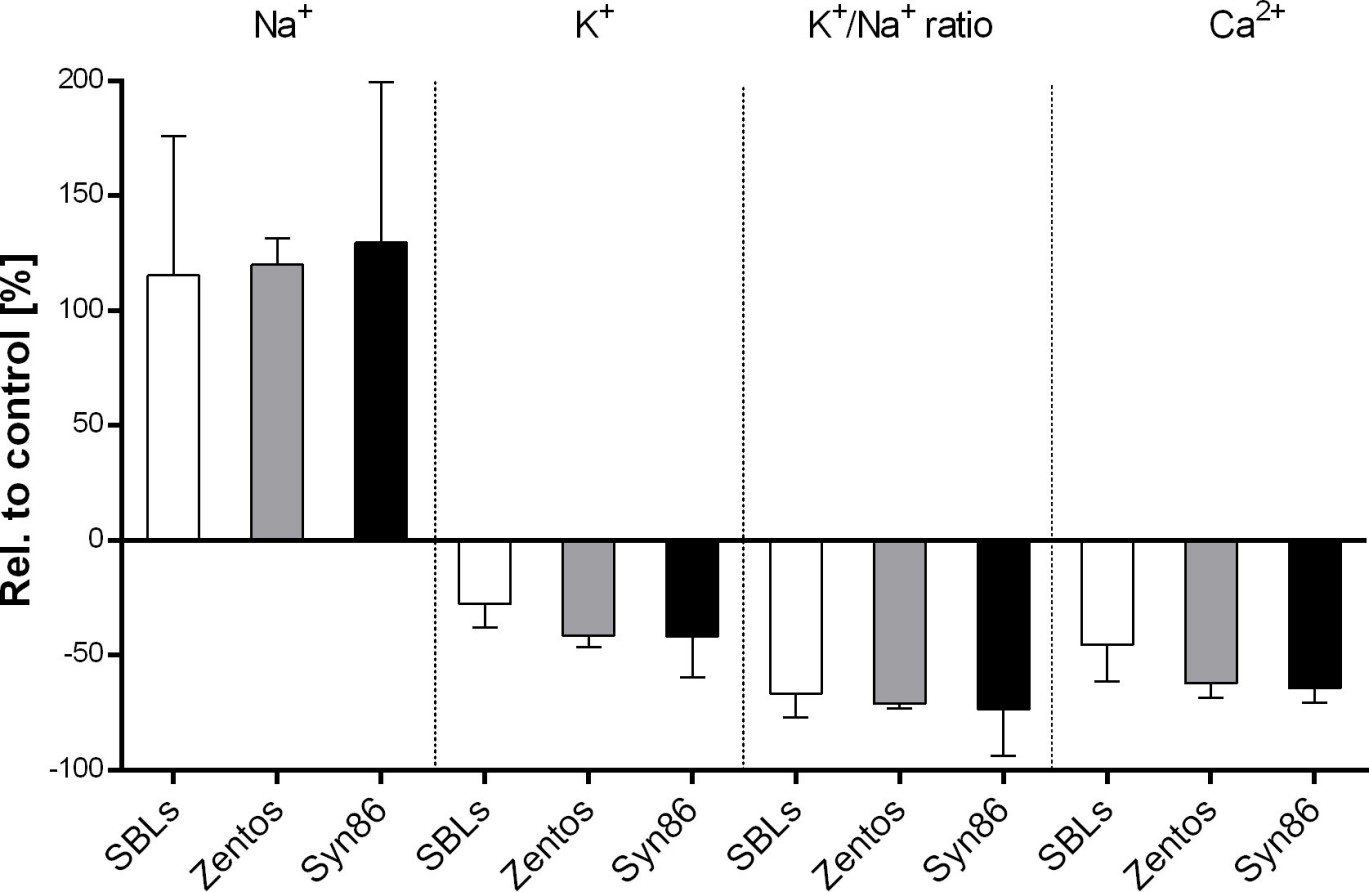
The concentration of Na^+^, K^+^, Ca^2+^ and K^+^/Na^+^-ratio in the third leaf of the Z86 population and their parents under 100 mM Na_2_SO_4_ relative to control; significant differences according to Tukey HSD (p ≤ 0.05) between treatments and groups are assigned with different letters.

To estimate the effect of anions and cations in response to stress, their contents in cytoplasm were measured (Fig 6). For that, the concentrations of Na^+^, K^+^ and Ca^2+^ of third leaves of the SBLs of the Z86 population and the parents, treated with 100 mM Na_2_SO_4_ compared to untreated plants, were analysed. Assessment of sodium concentration in third leaves of wheat plants revealed a two-fold higher concentration of Na^+^ ions in plants treated with 100 mM Na_2_SO_4_ compared to untreated plants. On the other side, the concentrations of the cations K^+^ and Ca^2+^ and the calculated Na^+^/K^+^ ratio were declining under salt stress conditions, although to a smaller extent. Under control conditions, the concentration of Na^+^ and K^+^ were showing positive but weak correlation (Figure F in S1 File). Under salinity stress conditions, this relation was negative. Also, there was no significant difference detectable between the parents and the progenies of the Z86 population.

### Phenotypic characterization at maturity stage

The performance of the SBLs of the Z86 population were evaluated under field conditions with natural salinization in Karshi (Uzbekistan) in three successive planting seasons from year 2010 to 2013. Assessment of most measured agronomic and morphological traits revealed moderate to high genotypic variation among the SBLs of the Z86 population under field conditions (under controlled as well as under salt stress conditions). Treatment effects were observed for most traits including grain quality parameters. However, G*T interaction effects were not observed for the measured traits (Table **7**).

**Table 7:** Descriptive statistics and analysis of variance explaining genotype (G), treatment (T) and genotype by treatment (G*T) interaction effects for parameters of the Z86 population measured under field conditions on saline and non-saline soils.

		Control			Saline condition			Genotype (G)		Treatment (T)		G*T		H^2^
Parameter		Mean	SD	CV	Mean	SD	CV	F-values	df	*F*-values	df	*F*-values	df	[%]
**DHD**		167.0	1.1	0.7	158.6	12.8	8.1	0.06 ^ns^	150	90.90***	1	0.05^ns^	150	10.0
**PH**		115.9	11.5	10.0	95.4	12.8	13.4	1.07 ^ns^	150	518.99***	1	0.96^ns^	150	13.7
**PL**		42.8	5.5	12.8	42.5	5.6	13.2	0.05 ^ns^	150	2.69***	1	0.01^ns^	150	98.6
**LS**		10.9	2.1	19.6	11.3	2.3	20.2	1.23 ^ns^	150	5.58*	1	0.75^ns^	150	31.2
**SpS**		20.5	2.5	12.0	20.5	2.5	12.3	1.07 ^ns^	150	518.99***	1	0.96^ns^	150	13.7
**TKW**		30.6	3.3	10.8	24.4	3.7	15.2	2.72***	150	563.85***	1	0.72^ns^	150	46.2
**Yield [T/ha]**		1.96	0.5	34.8	1.96	0.5	47.4	0.14^ns^	150	1.23*	1	0.78^ns^	150	23.3
**Grain quality**														
	Ash [%]	1.71	0.1	3.7	1.76	0.1	3.3	1.43**	150	105.67***	1	0.80^ns^	150	27.3
	Moisture [%]	10.21	0.95	8.3	8.89	0.2	2.5	0.81^ns^	150	460.25***	1	0.51^ns^	150	23.8
	Hardness [%]	58.74	3.2	5.5	56.54	3.1	5.5	3.18***	150	98.05***	1	0.69^ns^	150	64.9
	Protein [%]	18.08	2.3	12.9	20.62	1.9	9.4	1.21^ns^	150	184.85***	1	0.77^ns^	150	30.8
	Starch [%]	68.00	2.7	3.9	64.76	2.6	4.0	1.31*	150	208.43***	1	0.73^ns^	150	38.2
	Fiber [%]	2.70	0.2	7.7	2.63	0.2	8.6	3.68***	150	14.79***	1	0.89^ns^	150	59.9
	NDF [%]	18.60	1.2	6.5	18.45	1.1	6.0	1.91***	150	2.63^ns^	1	1.13^ns^	150	28.4
	Sedimentation [ml]	58.10	11.4	19.7	70.22	9.1	12.9	2.24***	150	233.36***	1	0.87 ^ns^	150	61.6

Note

Significance levels p

* p ≤ 0.05

** p ≤ 0.01

*** p ≤0.001

ns not significant; treatment refers to experimental plots on saline and non-saline soils, SD: standard deviation, CV: coefficient of variation, H^2^: broad sense heritability; DHD: days to heading; PH: plant height; PL: length of peduncle; LS: length of spike; SpS: spikelet per spike; TKW: thousand kernel weight; NDF: neutral detergent fibre.

Population-wide, minor reduction was observed for grain yield under salt stress compared to controlled conditions. Higher reduction induced by salt stress was detected for TKW (-20%) and starch content of harvested grains (-6%), whereas grain protein content increased under salinity stress (+14%) in comparison to controlled conditions (Table 7, Figure D in S1 File). Additionally, under salt stress conditions, plants had on average 18% reduced plant height as compared to control conditions.

Low correlation was detected for most morphological parameters measured in field trials under controlled and salt stress conditions (Table 8). Under control conditions, only PH was showing moderate correlation to length of peduncle (r = 0.4) whereas under salt stress conditions, a moderate positive correlation was found between grain yield and TKW (r = 0.4) and length of spike with number of spikelets per spike (r = 0.4). Likewise, higher correlation patterns (r >0.8) were observed between the grain quality traits measured with the NIR (Near Infra-Red) sensor under salt stress conditions in comparison to control conditions.

**Table 8:** Pearson's correlation coefficient (r) for parameters measured from Z86 population tested in field trials under natural salinization.

	Traits		DHD	PH	PL	SL	SpS	TKW	Yield	Grain quality								
										Ash	Mois.	Hard.	NDF	Prot.	Star.	Fibre	Sed.	
**Control**	**DHD**			0.171*	0.017	-0.032	-0.106	-0.112	0.108	-0.037	0.038	-0.059	0.101	-0.055	0.046	0.068	-0.112	**Stress**
	**PH**		-0.0		0.079	0.012	0.041	0.055	0.227**	-0.063	0.181*	0.090	-0.043	-0.035	0.044	0.019	0.002	
	**PL**		-0.104	0.396**		0.138	0.031	0.011	0.142	0.006	-0.095	0.143	-0.175*	0.129	-0.120	-0.017	0.259**	
	**SL**		0.015	0.211*	0.085		0.406**	0.089	0.132	-0.115	0.183*	0.056	-0.069	-0.061	0.098	-0.082	-0.007	
	**SpS**		0.060	-0.017	0.002	0.031		0.049	-0.125	-0.022	0.087	0.000	0.116	-0.039	0.023	-0.031	-0.024	
	**TKW**		-0.008	0.297**	0.170*	0.138	0.029		0.392**	-0.446**	-0.044	0.606**	-0.377**	-0.384**	0.453**	-0.651**	0.010	
	**Yield**		-0.165*	0.270**	0.210**	-0.099	0.011	0.308**		-0.393**	0.062	0.312*	-0.216	-0.322	0.375**	-0.407**	-0.165	
	**Grain quality**	**Ash**	-0.027	0.005	0.082	-0.079	-0.008	-0.194*	-0.114		-0.362**	-0.64**	0.319**	0.923**	-0.93**	0.686**	0.575**	
		**Mois.**	-0.019	0.076	-0.040	0.133	-0.072	0.203*	0.025	-0.094		0.071	-0.017	-0.34*0	0.406**	-0.003	-0.294	
		**Hard.**	0.138	0.143	0.1000	-0.078	-0.016	0.445**	0.197*	-0.385**	0.188*		-0.434**	-0.512**	0.610**	-0.829**	0.110	
		**NDF**	-0.081	-0.352**	-0.161	0.003	-0.007	-0.166*	-0.074	0.362**	-0.113	-0.473**		0.156	-0.27**	0.304**	-0.237**	
		**Prot.**	0.002	0.028	0.167*	-0.060	0.019	-0.193*	-0.143	0.902**	-0.222**	-0.293**	0.256**		-0.95**	0.645	0.770**	
		**Star.**	-0.007	0.028	-0.145	0.124	0.004	0.322**	0.205*	-0.903**	0.307**	0.444**	-0.364**	-0.940**		-0.739**	-0.623**	
		**Fibre**	-0.017	-0.016	0.003	-0.035	-0.040	-0.360**	-0.164*	0.441**	0.456**	-0.526**	0.160	0.312**	-0.7**		0.151	
		**Sed.**	0.091	0.138	0.242**	-0.095	0.042	0.043	-0.059	0.676**	-0.178*	0.233**	-0.042	0.844**	-0.6**	-0.027		

Note: DHD: days to heading, DMD: days to maturity, PH: plant height, PL: length of peduncle, SL: length of spike, SpS: spikelets per spike, TKW: 1000 kernel weight, Yield: (T/ha), Hard.: kernel hardness, NDF: Neutral Detergent Fiber, Prot.: protein content, Star.: starch content, Sed.: sedimentation

Significance levels p

* p ≤ 0.05

** p ≤ 0.01.

**Fig 7:**
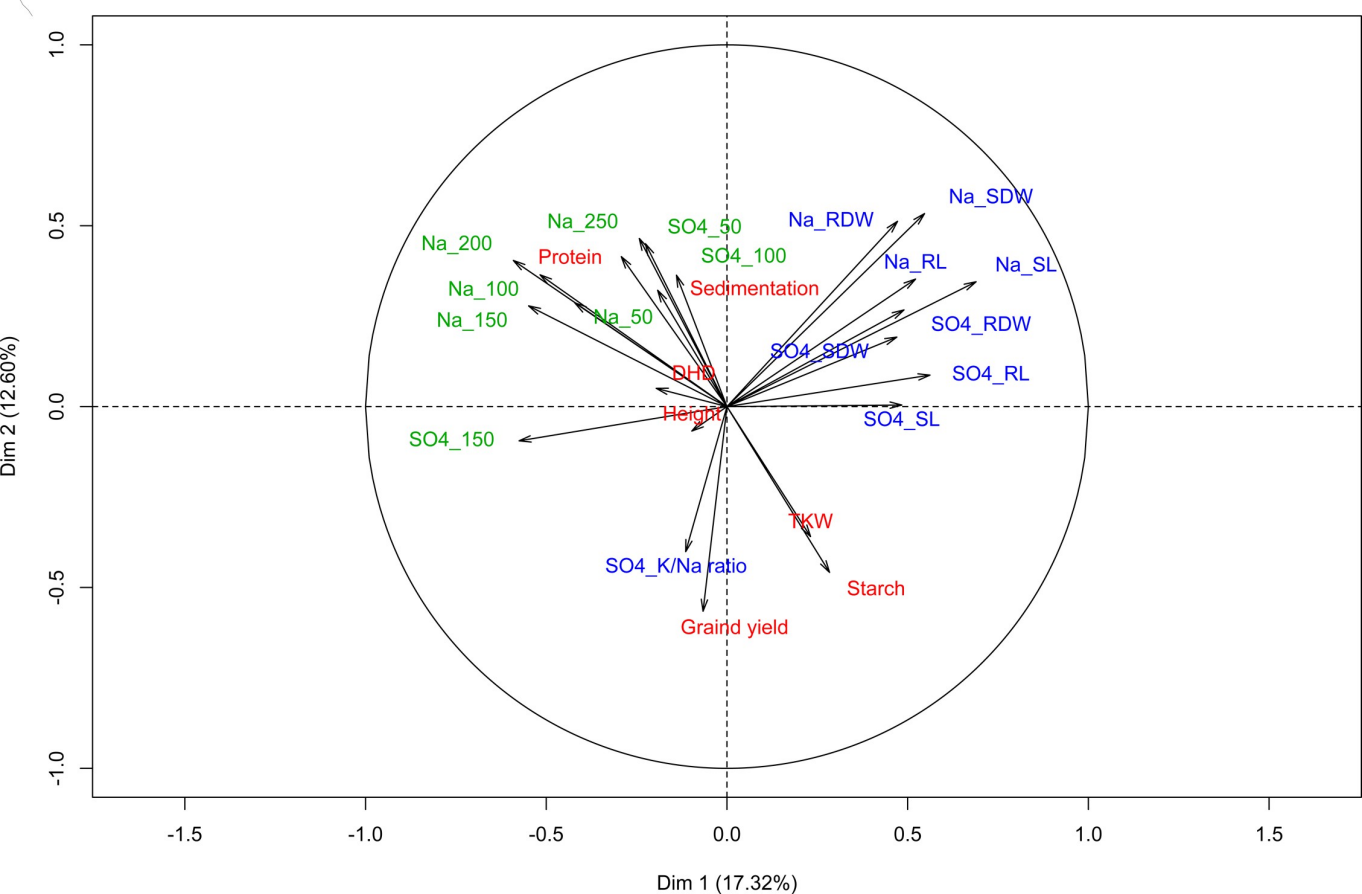
Principal component analysis(PCA) of major traits measured at three developmental stages of the Z86 population under salt stress conditons; Na_50 to Na_250: 50 to 250 mM NaCl; SO4_50 to SO4_150: 50 to 150 mM Na_2_SO_4_; SDW: shoot dry weight; RDW: root dry weight; SL: shoot length; RL: root length; DHD: days to heading; TKW: 1000 kernel weight; prefix “Na_” stands for treatments with NaCl salt and “SO4_” stands for treatments with Na_2_SO_4_ salt. Colors of parameters indicate germination stage (green), seedling stage (blue) maturity stage/field trials (red).

Yield performance and quality characteristics are essential demands to select appropriate varieties to cultivate under certain environmental conditions. However, the response of the tested genotypes to salt stress varied with development stage. Furthermore, different salt types and concentrations produced differential response depending on plant growth stage. However, according to the field trials under natural salinization, genotype WW34-33 was the most susceptible line (-54%) and genotype WW43-27 was the most salt tolerant line (+82%) (Figure E in S1 File). Moreover, weak and, in most cases, negative correlation was estimated for the measured parameters across growth stages. Moderately stronger and positive correlation were estimated for the traits of the same growth stage (Figure F in S1 File). Principal component analysis (PCA) was performed to visualize the relationship among major traits evaluated at three growth stages (Fig 7). The PCA plot is indicating a stronger correlation between K^+^/Na^+^-ratio at seedling stage and grain yield under field conditions. However, no correlation was observed between K^+^/Na^+^-ratio and other traits at seedling stage.

## 4. Discussion

Above all abiotic stress factors, drought and salinity stress have the highest impact on global wheat productivity and pose a major challenge to food security [63, 64]. Genetic variability is a fundamental requirement for breeding salt tolerant crops which is regarded as one of the most promising options to close the food gap imposed by salinity stress [45, 65]. The AB-population Z86 derived from hybridizations between the elite winter wheat cultivar Zentos and the SHW Syn86L has proven to be a valuable population for the identification and selection of improved germplasm lines for breeding cultivars with improved baking quality [50] and biotic stress tolerance [66]. In the present study, the Z86 population was evaluated under various salt treatments at different growth stages for detection of SBLs with higher salinity tolerance. Genetic variability among the tested genotypes was evident for most traits through all growth stages. At germination stage, in agreement with previous reports, higher concentrations of applied salts adversely affected seed germination. The detrimental effect of the equal concentration of Na_2_SO_4_ was approximately two-fold higher than the same concentration of NaCl as previously reported [27, 67, 68]. At the germination stage, the parents exhibited difference response to salinity with Zentos showing higher germination rates than Syn86 across all salt types and concentrations.

The importance of germination stage in plants withstanding salinity stress has previously been discussed [35, 69, 70]. Other authors suggest germinations tests as an ideal approach for evaluations of a large number of genotypes for salt tolerance at early growth stages [13, 33, 71–73]. Based on the germination tests, 22 SBLs (15% of the population) showed positive mid-parental transgressive segregations (MTS) at 200 mM NaCl and two genotypes, at highest NaCl concentration of 250 mM. At 100 mM Na_2_SO_4_, only 12 SBLs exhibited MTS whereas one (genotype WW34-49) showed tolerance at the highest Na_2_SO_4_ concentration of 150 mM. Backcross lines of various crops showing transgressive segregations for salinity tolerance at germination stage was previously reported by several authors [54, 74–76]

Hydroponic systems have become a standard approach for evaluation of plants for salinity stress at seedling stage [6, 38, 56]. The advantages of aerated hydroponic system established within this study enable to phenotype efficiently a large number of plants. Major advantages of this system are its easy handling, fast maintenance and low cost. Additionally, different to the system described by [35] and Genc et al. [6], the aerated hydroponic system has advantages due to its flexibility, and enabling quick *in-vivo* measurement of root length with minimal disturbance of the plants. Like at the germination stage, high phenotypic variation of the SBLs and their parents was observed at seedling stage in hydroponics under control conditions as well as under 100 mM NaCl or Na_2_SO_4_.

As the roots of the plants were in direct contact with the high concentrations of NaCl and Na_2_SO_4_, respectively, the reduction of RL for all genotypes was stronger than the reduction of SL. Measurements of root elongation rate (RER) and shoot elongation rate (SER) revealed genotype by treatment interactions for RER. Compared to control conditions, REL of Syn86 was more reduced during the first phase (Δ1) whereas RER of Zentos exhibited the most reduction during Δ2. Notably, the SER of Zentos was outperforming Syn865L and the SBL mean. Munns et al. [77] reported similar results when they investigated elongation rates of leaves of barley and maize plants exposed to salinity shock with and without excised roots. They observed a brief decline followed by a quick recovery of elongation rate in plants with excised roots in contrast to plants with intact roots. Obviously, root length has a high impact on the level of impairment induced by salinity stress. Plants with shorter roots and lower root surface might have an advantage in comparison with plants with longer roots and greater root surface since they have a comparably smaller root area which is in contact with the deleterious solution.

Similar to the results obtained at germination stage, at seedling stage, the elite parent Zentos outperformed the synthetic parent Synh6L with respect to most biomass related traits like SDW and RDW. Additionally, several genotypes surpassed their parents for SDW, RDW and other major traits, revealing positive mid-parental transgressive segregations.

However, for RL and SL, the SHW Syn86L outperformed the elite cultivar Zentos and most of their progenies at seedling stage under control as well as under salt stress conditions. Several studies with landraces, wild or exotic relatives of adapted crops support this observation with respect to SL [78, 79] and RL [80–82]. As reviewed by Ogbonnaya et al. [47], these exotic and advantageous traits were subject of multiple studies and breeding approaches for introgression of these alleles into modern cultivars to enhance their performance by pyramiding favourable alleles. Accordingly, by application of QTL analysis with a backcross population, Uga et al. [83] were able to localize the DEEPER ROOTING 1 (DRO1) allele which controls root growth angle descending from a wild rice genotype. By backcrossing the DRO1 containing introgression line with a shallow-rooting rice, the authors were able to confirm the methodology to introgress beneficial exotic alleles from wild genotypes into modern cultivars to enhance their abilities under adverse environmental conditions.

Salinity tolerance is frequently attributed to ability of plants to maintain a high K^+^/Na^+^ ratio by discrimination of Na^+^ and preferential uptake of K^+^ [84, 85]. However, ionic analysis of third leaves of Syn86L and Zentos of plants exposed to 100 mM Na_2_SO_4_ did not show significant differences with respect to K^+^ and Na^+^ concentrations and K^+^/Na^+^ ratio. No correlation was detected between SDW at seedling stage and concentrations of analysed mineral components Na^+^, K^+^ and Ca^2+^ and the K^+^/Na^+^ ratio in third leaves of plants exposed to 100 mM Na_2_SO_4_. Genc et al. [6] reported similar observations assessing the salinity tolerance of 21 bread wheat genotypes, proposing that salinity tolerance in some genotypes might be driven by tissue tolerance rather than Na^+^ exclusion or a combination of both mechanisms. However, results from this study revealed a strong correlation between K^+^/Na^+^ ratio at seedling stage and grain yield under field conditons. Nevertheless, Munns et al. [13] found a Na^+^ discrimination and preferential uptake of K^+^ in salt tolerant genotypes relative to susceptible genotypes.

At germination stage, the parents Zentos and Syn86L behaved contrastingly compared to the seedling stage. Better performance of Zentos over Syn86L was detected at germination stage across all salt treatments for SDW and RDW. Obviously, this is in contrast to several studies endorsing synthetic hexaploid wheat as more tolerant than modern elite cultivars, making them ideal donors of exotic alleles contributing to tolerance towards biotic and abiotic stresses like salinity tolerance [86–88]. However, morphological and physiological variation among SHW and *Ae*. *tauschii* accessions, as their progenitor, was previously reported [49, 89]. According to Dubcovsky and Dvorak [90] and Li et al. [91], due to its genome plasticity, modern wheat is showing higher adaptation and robustness towards various environmental conditions, including salinity stress. Due to the efficient and long-term breeding process, modern wheat varieties not only increased their performance with respect to higher yield and quality traits, but also show a high-level robustness towards abiotic and biotic stress factors [91]. Still, the exotic and synthetic genotypes might contain favourable alleles that will display their advantageous trait effect if introgressed in a modern wheat genetic background.

The SBLs of the Z86 population showed high variation with respect to the measured parameters, including grain yield. However, in agreement with previous reports [35, 92, 93], the correlations between the selected parameters at the three developmental stages was weak. Several studies confirm the differential sensitivity of plants to salinity at different growth stages [30, 94, 95], expressing their concerns for the robustness of field experiments. They assume that especially grain yield of foreign or not adapted genotypes will be highly influenced by the environmental and exogenic stress factors. Therefore, the genotype or the genotype*treatment interaction effects might not be significant [13, 96, 97]. Additionally, evaluation of a large number of genotypes under saline conditions is difficult because of the variability of salinity within fields [98, 99]. This argumentation may also be applied to the field experiments that were conducted under continental climate zone conditions in Karshi (Uzbekistan), with high annual variation in temperatures between summer and winter seasons [100].

Moreover, SHW Syn86L employed in this study offered a higher genetic variability than the SHW used in many other studies [49, 101, 102]. Other than commonly used SHW, which are mainly based on hybridization between durum wheat (*Triticum durum* L.) and *Aegilops tauschii* (Coss.), Syn86L was produced by hybridizations of wild emmer (*Triticum turgidum* spp. *dicoccoides*) and *Ae*. *tauschii*.

In summary, the outcome of this study is supporting the hypothesis of Neumann [103], that root reduction might be an adaptive biophysical response of plants to cope with salinity stress. Vice versa, salt-tolerant plants with the efficient regulation of K^+^/Na^+^ homeostasis might be able to maintain longer roots, which is of importance for uptake of water and nutrients. However, this observation might be different in natural soil conditions since hydroponic systems are artificial systems where plants roots are continuously surrounded by the hydroponic solution.

However, our experiments revealed that for almost all traits, genotypes among the progenies of the Z86 population showed higher performance than their parents with some SBLs showing significant transgressive segregation for traits like SDW and RDW. Hence, these lines might provide useful germplasm for understanding the mechanisms of transgression in salt tolerance. On the other side, the integration of the advantageous SBLs carrying favourable alleles in breeding programs will help to breed improved high yielding and at the same time high quality wheat varieties withstanding severe salt stress conditions. Yet, supported by several studies, regardless of their performance, introgression of exotic alleles from SHW into modern cultivars by backcrossing approach will increase the probability to result in genotypes with enhanced trait expression due to gene interactions [89].

## 5. Conclusions

Introgression of exotic alleles from SHW into elite bread wheat cultivar by advanced backcrossing approach come to fruition of progenies with higher performance than the best performing parent with respect to the measured morphological and physiological parameters. Under natural field conditions, plants benefit from long roots, accessing deeper soil layer with lower salt concentrations. However, root length might be a drawback in hydroponic experiments inducing reduction of biomass under salinity stress by over-excess of Na^+^ intrusion into the plant. And the reduction of root length under salinity stress might be a long-term response of plants to cope with salinity by avoiding overload of Na^+^. However, few progenies were outperforming both parents with respect to salinity tolerance at germination stage and biomass production at seedling stage. Those genotypes indicate on introgressions of beneficial alleles descending from the exotic parent Syn86L. These genotypes might be considered as potential candidates for better understanding of salt stress tolerance mechanisms in plants and their application in breeding programs for efficiently breeding salt tolerant cultivars with superior grain quality traits.

## Supporting information

S1 File Supplementary figures. (DOCX)

## Abbreviations

BTS: Best-parental transgressive segregation
DAS: Days after stress initiation
DHD: Days to heading
MTS: Mid-parental transgressive segregation
PH: Plant height
PL: Peduncle length
RDW: Root dry weight
RL: Root length
SBL: Synthetic backcross lines
SDW: Shoot dry weight
SHW: Synthetic hexaploid wheat
SL: Shoot length
SPL: Spike length
SpS: Spikelets per spike
STI: Stress Tolerance Index
TKW: 1000 kernel weight.
