# Supplementary material for: Multi-dimensional evaluation of response to salt stress in wheat
Source: PLoS One. 2019 Sep 30;14(9):e0222659. doi: 10.1371/journal.pone.0222659 (PMC6768486; doi:10.1371/journal.pone.0222659)
Supplement: S1 File — (DOCX) [file pone.0222659.s001.docx]

**Supporting information**

**Figure A in S1 File.** Setup of the aerated hydroponic system for phenotyping of the Z86 population under control and salinity stress conditions

**
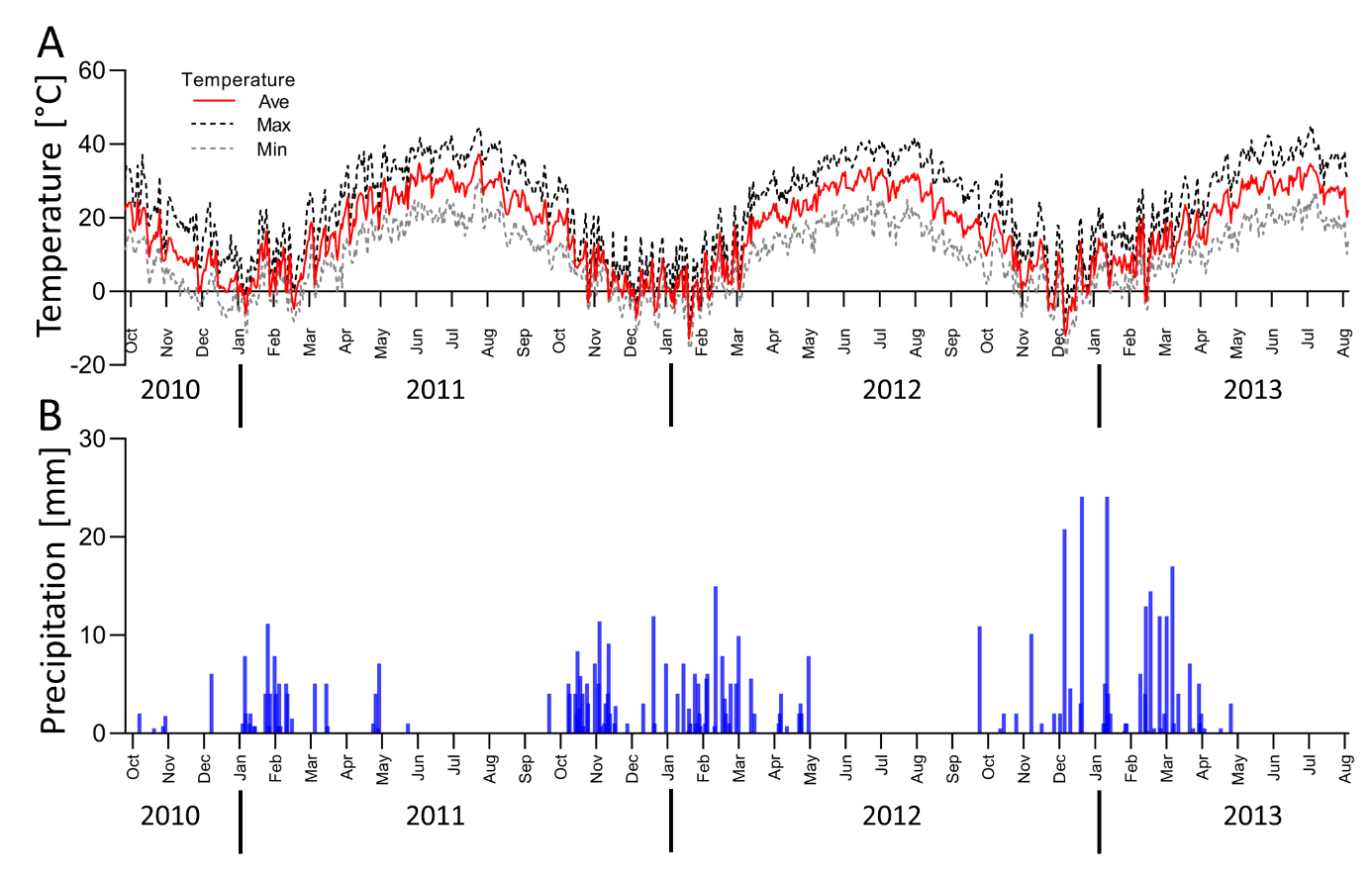
**

**Figure B in S1 File.** Average, maximum and minimum daily air temperature (A) and daily

precipitation (B) measured at the experimental site in Karshi from October 2010 to August

2013. (Source: www.noaa.gov)


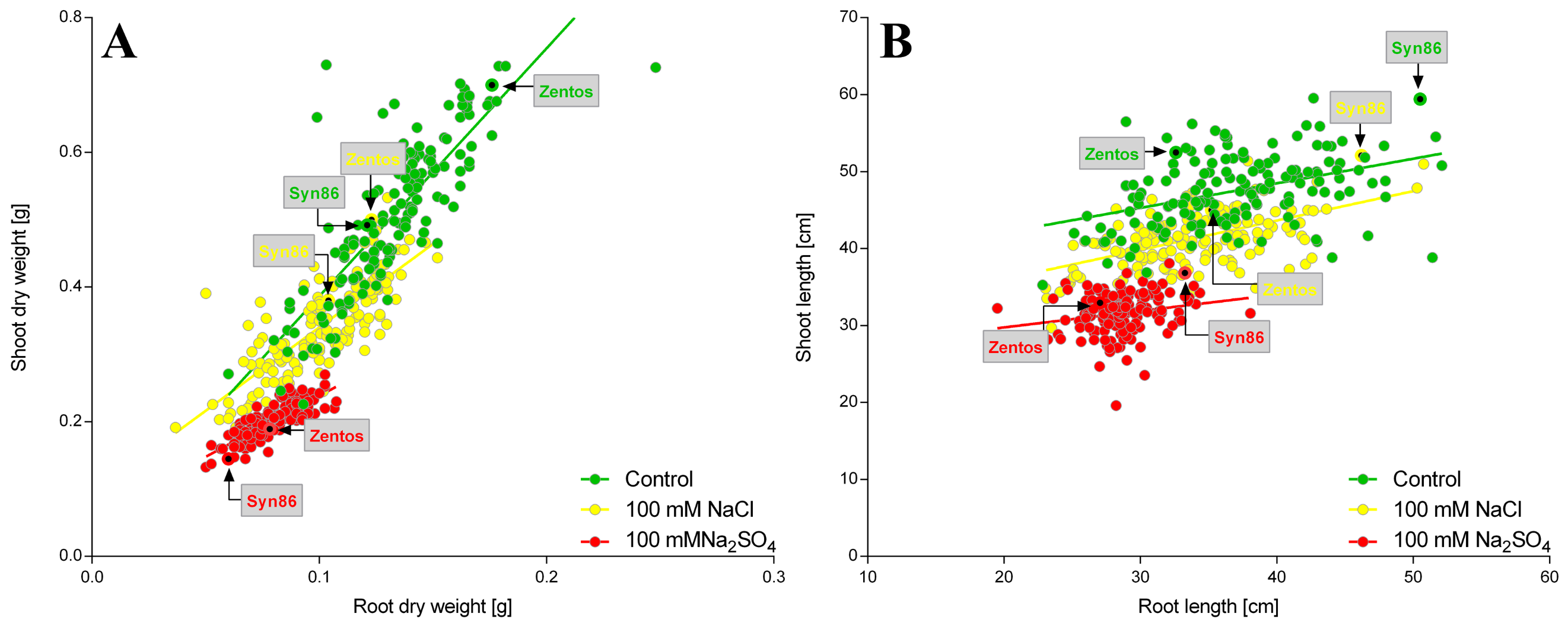


**Figure C in S1 File.** Shoot and root dry weights (A), and shoot and root length (B) of the Z86 population grown in hydroponic systems under control conditions (green), 100 mM NaCl (yellow) and 100 mM Na_2_SO_4_ (red)


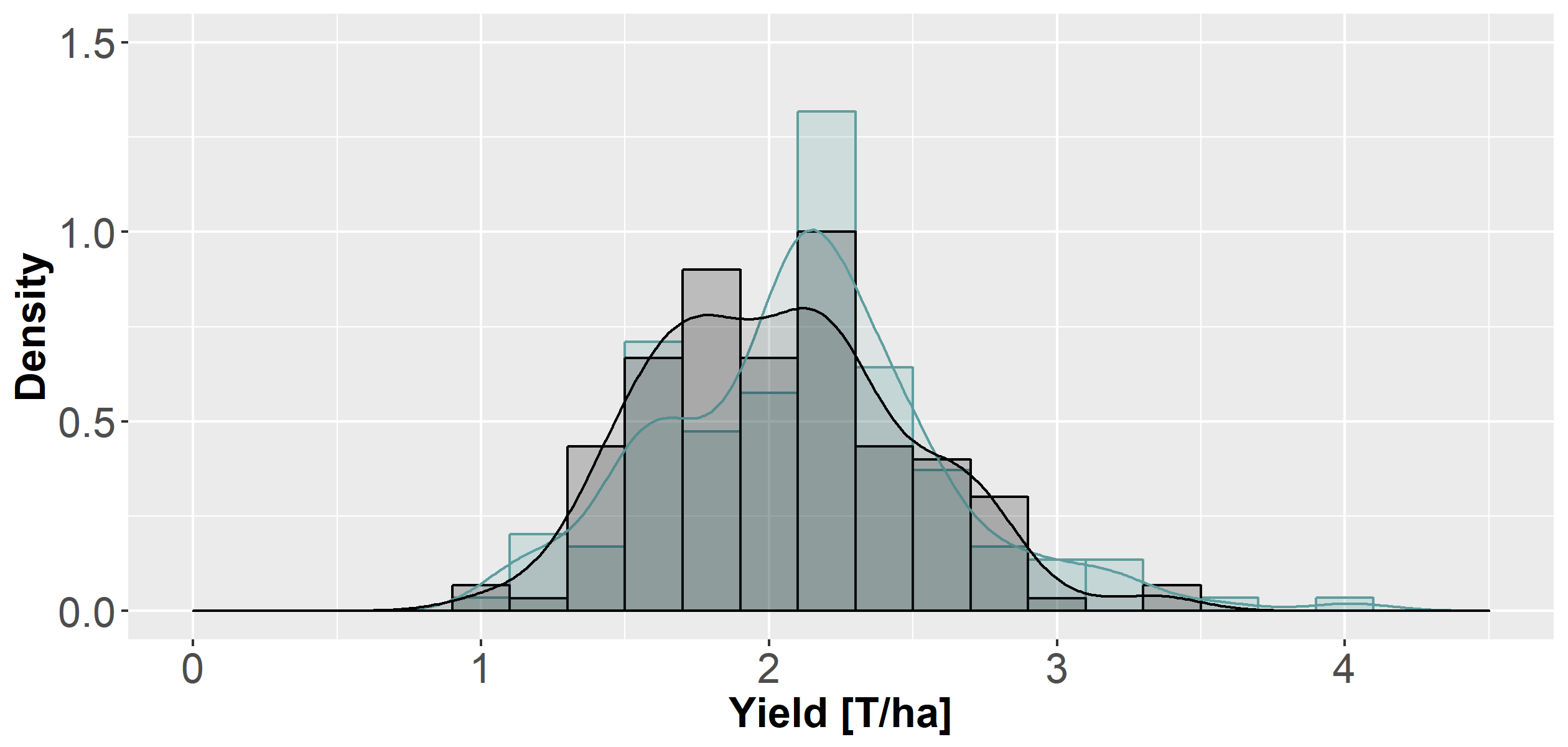


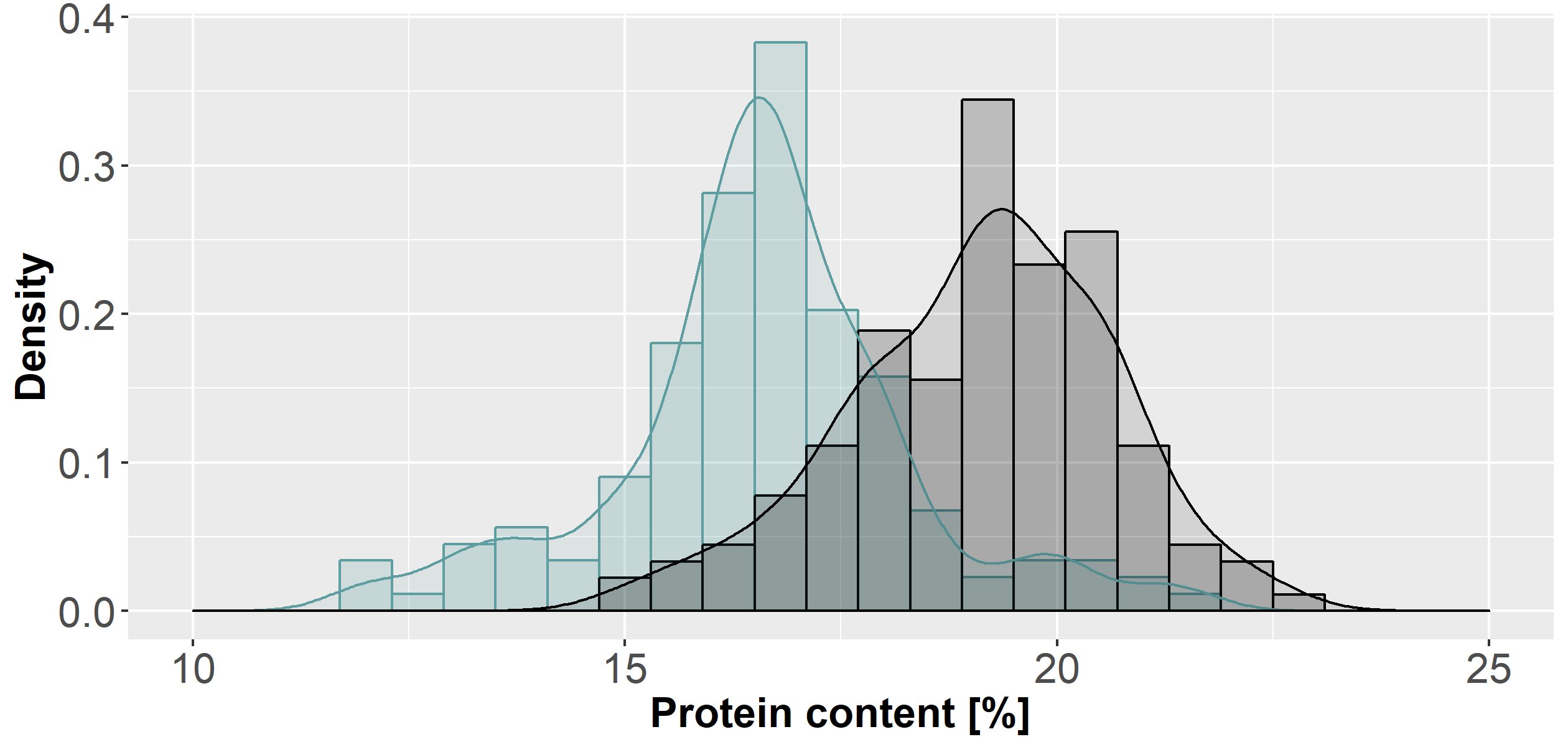

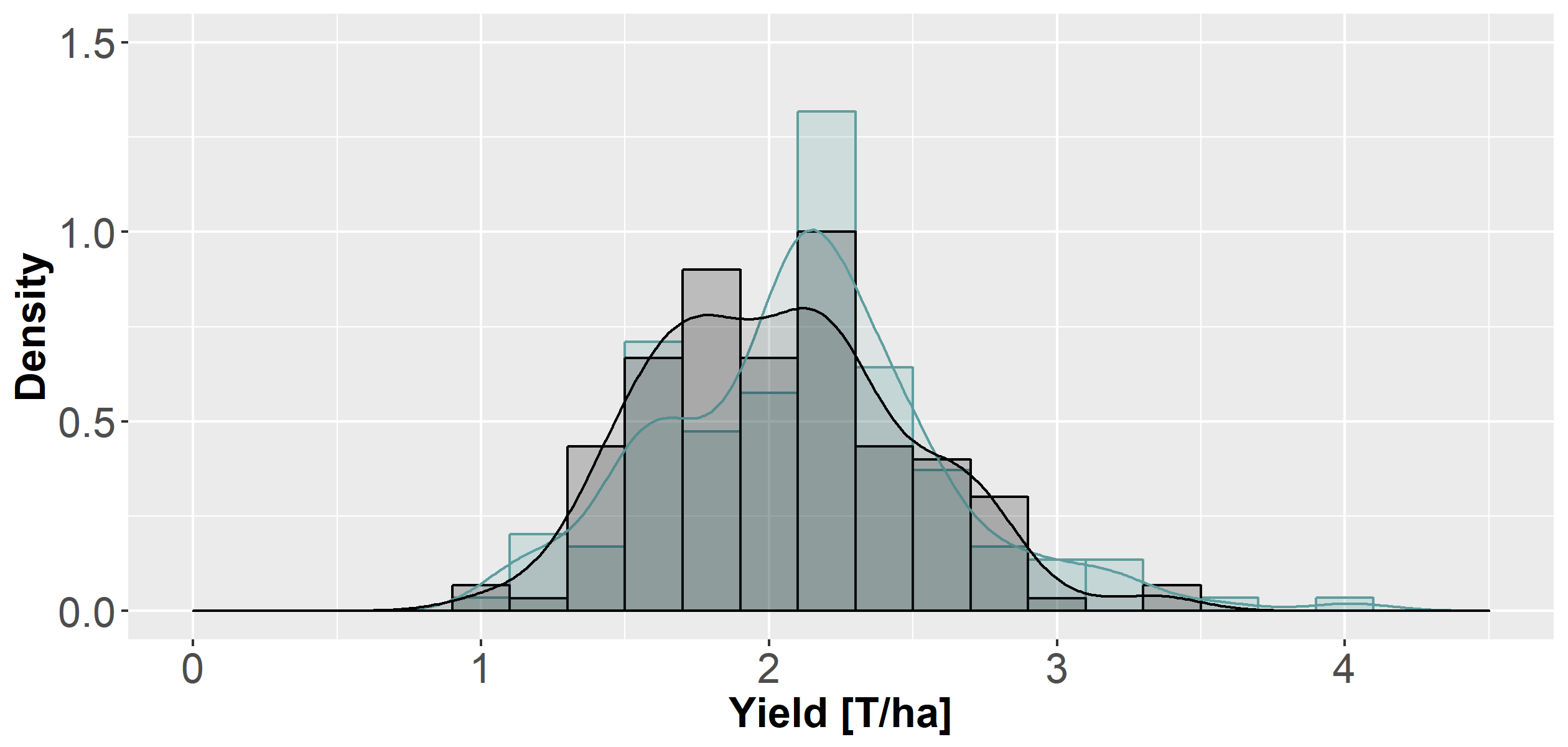

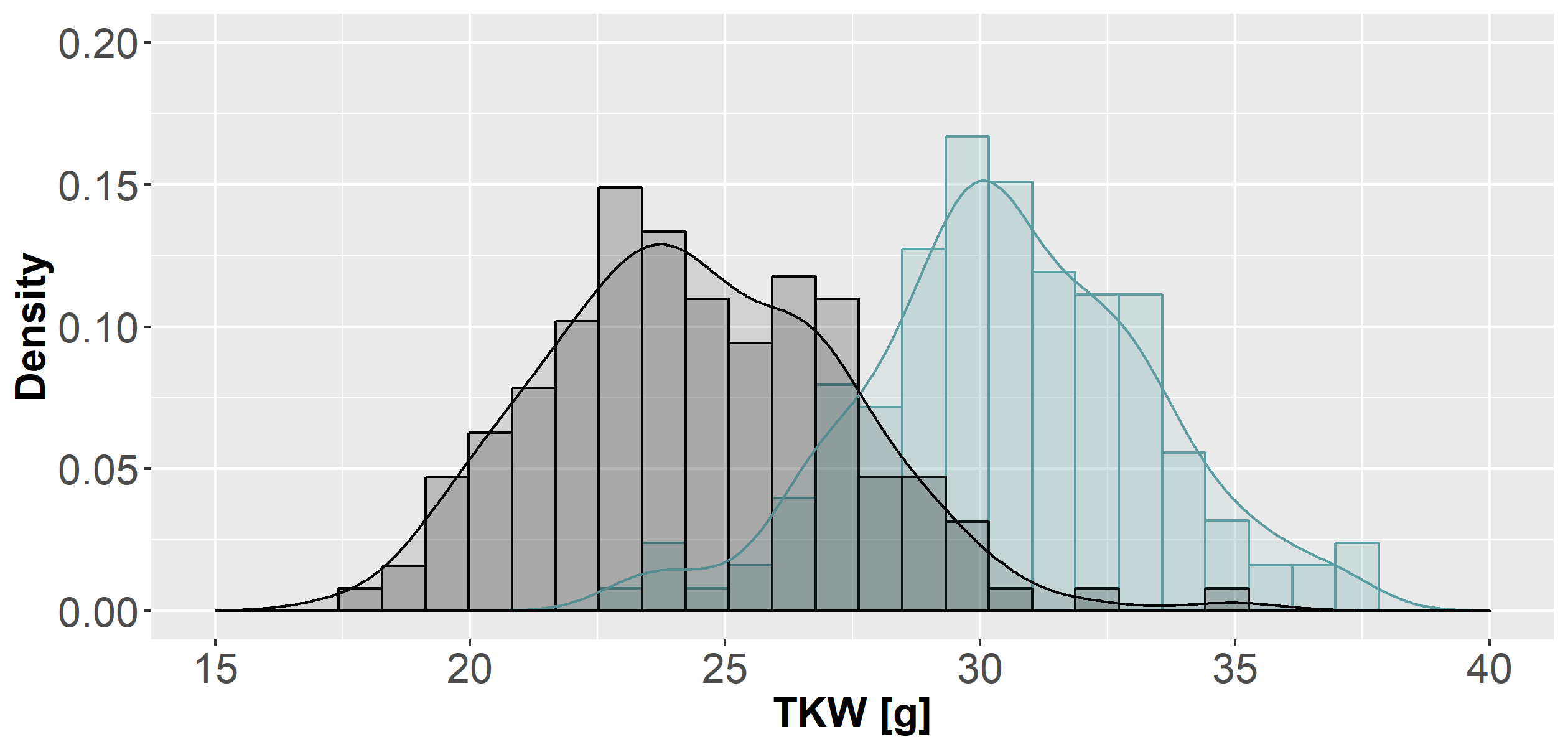

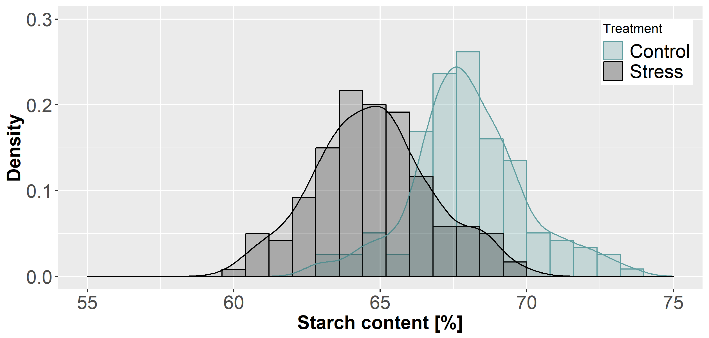


**Figure D in S1 File.** Density plots of grain yield, TKW (1000 kernel weight), kernel protein

and starch content of the Z86 population under field conditions


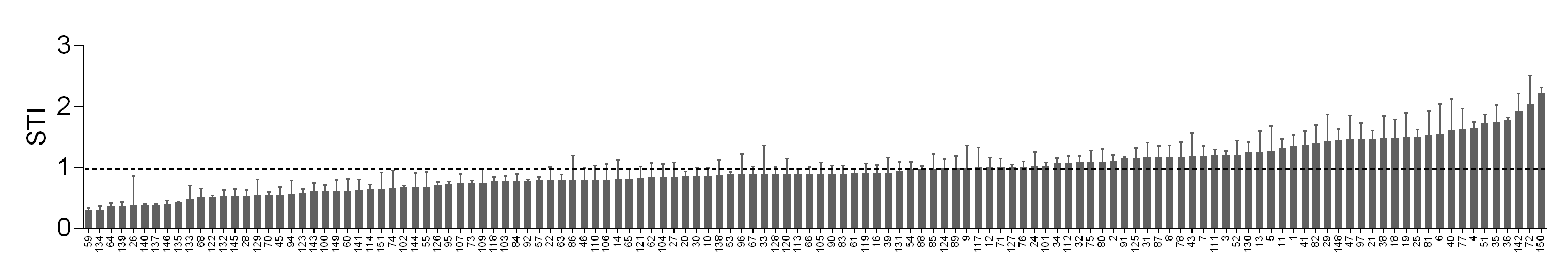


**Figure E in S1 File.** Grain yield of the lines of the Z86 under salt stress conditions compared to the population mean under salinity stress; error

bars indicate standard deviations; genotypes are sorted according the highest STI value

**
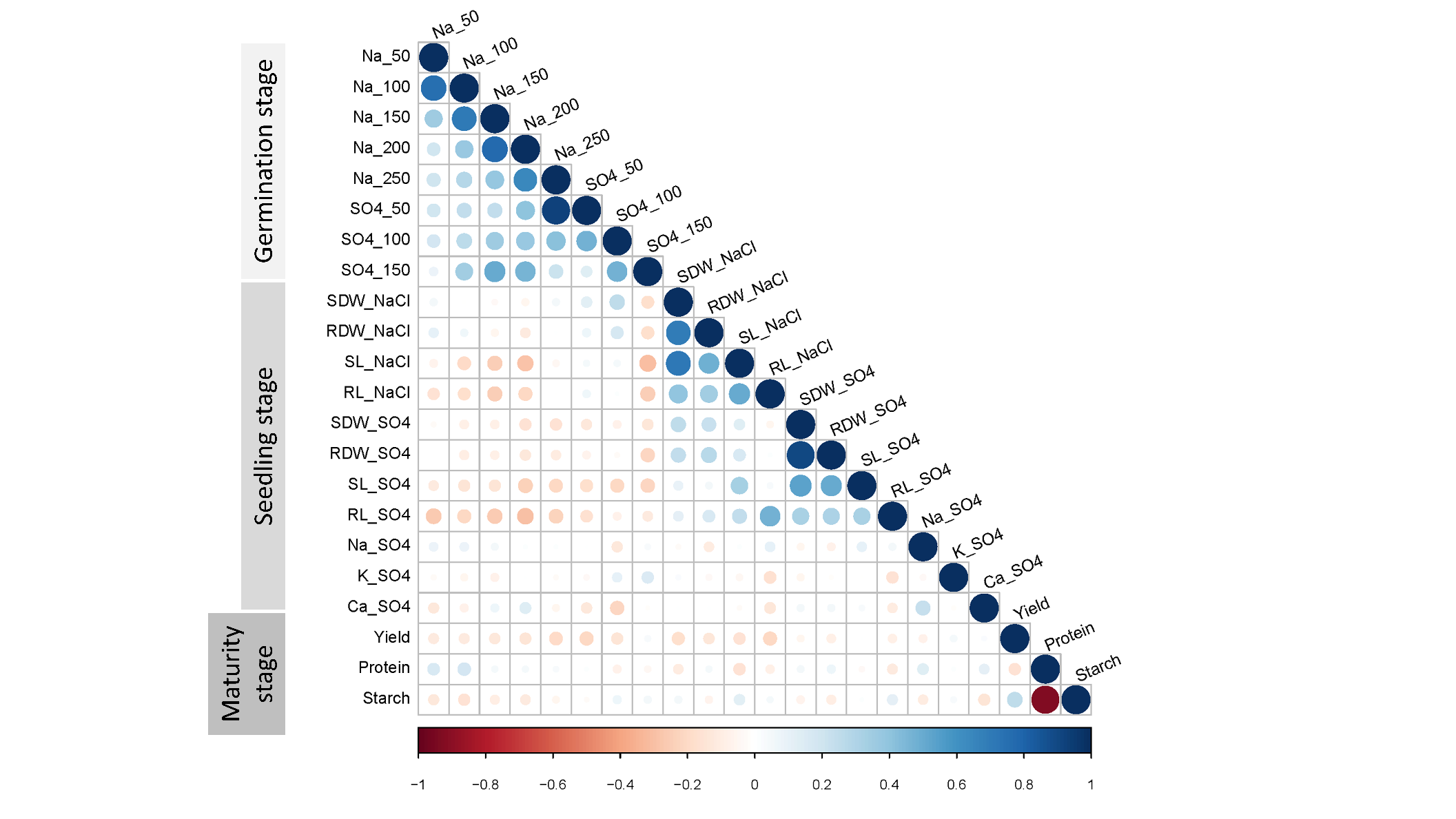
**

**Figure F in S1 File.** Correlation matrix of STI values of the major parameters of the Z86

population at germination, seedling and maturity stage

**Parameters at germination stage:** Na_50 to Na_250: 50 mM NaCl to 250 mM NaCl; SO4_50 to SO4_150: 50 to 150 mM Na_2_SO_4_.

**Parameters at seedling stage:** experiment with 100 mM NaCl; SO4: experiment with 100 mM Na_2_SO_4_; SDW shoot dry weight; RDW root dry weight; SL shoot length; RL root length; Na, K, Ca: concentration of Na^+^, K^+^ and Ca^2+^ in third leaves

**Parameters at maturity stage:** grain yield, grain protein and starch content.
